# Single Neurons in the Avian Auditory Cortex Encode Individual Identity and Propagation Distance in Naturally Degraded Communication Calls

**DOI:** 10.1523/JNEUROSCI.2220-16.2017

**Published:** 2017-03-29

**Authors:** Solveig C. Mouterde, Julie E. Elie, Nicolas Mathevon, Frédéric E. Theunissen

**Affiliations:** ^1^Equipe de Neuro-Ethologie Sensorielle-Neuro-PSI CNRS UMR9197, Université de Lyon/Saint-Etienne, 42023 Saint-Etienne, France, and; ^2^Helen Wills Neuroscience Institute, University of California, Berkeley, California 94720, and; ^3^Department of Psychology, University of California, Berkeley, California 94720

**Keywords:** auditory scene analysis, electrophysiology, ranging, songbird, sound propagation, vocal communication

## Abstract

One of the most complex tasks performed by sensory systems is “scene analysis”: the interpretation of complex signals as behaviorally relevant objects. The study of this problem, universal to species and sensory modalities, is particularly challenging in audition, where sounds from various sources and localizations, degraded by propagation through the environment, sum to form a single acoustical signal. Here we investigated in a songbird model, the zebra finch, the neural substrate for ranging and identifying a single source. We relied on ecologically and behaviorally relevant stimuli, contact calls, to investigate the neural discrimination of individual vocal signature as well as sound source distance when calls have been degraded through propagation in a natural environment. Performing electrophysiological recordings in anesthetized birds, we found neurons in the auditory forebrain that discriminate individual vocal signatures despite long-range degradation, as well as neurons discriminating propagation distance, with varying degrees of multiplexing between both information types. Moreover, the neural discrimination performance of individual identity was not affected by propagation-induced degradation beyond what was induced by the decreased intensity. For the first time, neurons with distance-invariant identity discrimination properties as well as distance-discriminant neurons are revealed in the avian auditory cortex. Because these neurons were recorded in animals that had prior experience neither with the vocalizers of the stimuli nor with long-range propagation of calls, we suggest that this neural population is part of a general-purpose system for vocalizer discrimination and ranging.

**SIGNIFICANCE STATEMENT** Understanding how the brain makes sense of the multitude of stimuli that it continually receives in natural conditions is a challenge for scientists. Here we provide a new understanding of how the auditory system extracts behaviorally relevant information, the vocalizer identity and its distance to the listener, from acoustic signals that have been degraded by long-range propagation in natural conditions. We show, for the first time, that single neurons, in the auditory cortex of zebra finches, are capable of discriminating the individual identity and sound source distance in conspecific communication calls. The discrimination of identity in propagated calls relies on a neural coding that is robust to intensity changes, signals' quality, and decreases in the signal-to-noise ratio.

## Introduction

One of the biggest tasks for the brain is to discriminate, in the midst of the prodigious amount of stimuli that it continuously receives, what is relevant from what is not. This task is further constrained by the multiple sources of noise in natural environments that contribute to the degradation of the biologically relevant information. Although real-world “scene analysis” is a universal problem solved by all animals (Schnitzler and Flieger, 1983; Aubin and Jouventin, 2002; von der Emde, 2004; Appeltants et al., 2005), understanding this process is a challenge for scientific research (Bregman, 1993). A major limitation in current neurophysiological approaches is that they do not address the complexity of the problem (Lewicki et al., 2014): experiments using “idealized” stimuli that are often not ecologically relevant have little chance of providing a thorough understanding of the mechanisms at play in natural settings. In this respect, one overlooked aspect in auditory scene analysis concerns how the brain extracts socially relevant information when acoustic signals are degraded by long-range propagation through the environment. Focusing on a songbird model, whose acoustic communication demonstrates prominent similarities to human speech (Doupe and Kuhl, 1999), we investigated the neural substrate allowing discrimination between individual vocal signatures despite extreme propagation constraints.

The zebra finch (*Taeniopygia guttata*) is a social songbird from sub-arid Australia that uses a particular contact call, the distance call, to establish contact at a distance with groupmates or family members (Zann, 1996; Mulard et al., 2010). This short call is emitted by both sexes, bears an individual signature, and pair-bonded adults use it to both identify and locate their partner when a visual connection has been lost (Zann, 1984, 1996; Vignal et al., 2004, 2008). While long-range propagation through the environment induces a profound degradation of the calls' quality (Wiley and Richards, 1982; Mouterde et al., 2014b), previous experiments showed that female zebra finches can still discriminate between the calls of males propagated at distances >100 m (Mouterde et al., 2014a). This impressive perceptual ability must be mediated by auditory neurons whose responses, coding for identity information, are only minimally affected by propagation-induced sound degradations.

A previous study investigating the neural basis of discrimination between conspecific songs in the zebra finch brain found intensity invariant neurons in the Field L, a region analogous to the primary auditory cortex in mammals (Billimoria et al., 2008). Other studies indicated an increased tolerance for masking noise as one ascends the auditory pathway, describing noise-invariant neurons in the caudomedial nidopallium (NCM), a secondary auditory area (Boumans et al., 2008; Moore et al., 2013; Schneider and Woolley, 2013). However, in all of these studies, the acoustical quality of the vocalizations was preserved; we do not know how the songbird brain deals with the impact of propagation-induced degradations as naturally experienced by the animals when communicating at long range. These sound degradations encompass the joint effects of sound intensity decrease, background noise, and spectrotemporal alterations of the signal (Forrest, 1994). Individual discrimination should be supported by neurons invariant to all of these degradations.

In addition, the estimation of the signaler's distance through the perception of distance-dependent degradations of vocalizations is a ranging ability well demonstrated in songbirds (Naguib and Wiley, 2001; Mathevon et al., 2008). Whereas distance-invariant neurons are expected for individual discrimination, ranging should rely on distance-sensitive neurons. However, it is unclear how the identity and the distance of the vocalizer are extracted from degraded signals by the auditory system and encoded in neuronal responses. For example, we do not know whether identifying and localizing a vocalizer involve different neuronal populations within the songbird auditory areas.

Here, we quantified, throughout the zebra finch primary and secondary brain auditory areas, the discrimination and coding properties of single neurons for both the individual vocal signature and the propagation distance of calls propagated in natural conditions.

## Materials and Methods

### 

#### 

##### Stimulus design and recordings.

The stimuli included natural and synthetic calls of male and female zebra finches. The *Natural* stimuli were distance calls from unrelated and unfamiliar conspecifics to the subjects, which had been propagated at various distances (from 2 to 256 m) and recorded in natural conditions. The *Natural* stimuli tested the overall effect of the decrease of the signal-to-noise ratio (SNR), induced by the transmission through the environment, on neural responses. SNR is here defined in the broad sense (i.e., taking into account both the effects of the decrease of the signal's amplitude comparatively to the relatively constant amplitude of the background noise as well as the degradation of the call's temporal and spectral structure due to propagation through the environment). To isolate the sheer effect on neural responses of signal intensity decrease, due to sound spherical spreading and excess attenuation, neural responses to *Synthetic* stimuli were also recorded. In the *Synthetic* stimuli, the sound intensity matched that of the *Natural* calls at each propagation distance, but the SNR was constant (see details below).

To prepare the *Natural* stimuli, we used a database of distance calls recorded from 32 zebra finches (16 females, 16 males) raised in the Equipe de Neuro-Ethologie Sensorielle Laboratory. The calls were recorded in a soundproof room using a microphone (Sennheiser MD-42) placed 0.2 m above the cage and connected to a Marantz Professional Solid state recorder (PMD 670; sampling frequency 44,100 Hz). Each bird was recorded in the presence of two females placed 3 m away and used as an audience to minimize stress; the bird was stimulated with distance calls playbacks from previously recorded conspecific birds. These experimental protocols were approved by the Jean Monnet University's animal care committee (authorization 42-218-0901-38 SV 09 to Equipe de Neuro-Ethologie Sensorielle Laboratory). We recorded 16 different calls from each individual to make our call database (total number of calls: 16 × 32 = 512 calls). The intensity of all the calls was normalized by matching the maximum values of the sound pressure waveforms. We recorded the propagated sounds from all the calls of this database in natural conditions on an open flat field. For these sound recordings, the calls were broadcast from a portable solid-state recorder (Marantz PMD671) connected to a MegaVox speaker (PB-35W) placed on a stool, 1.30 m high. The speaker volume was set to obtain a sound level of 70 dB SPL at 1 m (Vignal et al., 2008). The sounds were recorded with a Schoeps microphone (MK4 cardioid, on a CMC6-U base) equipped with a Schoeps Basket-type Windscreen (W20) and set 1.30 m high. The microphone was connected to a second Marantz recorder (PMD671; sampling frequency 44,100 Hz). The propagated calls were recorded 2, 16, 64, 128, and 256 m away from the source. From these recordings, we isolated 16 different calls per individual per propagation distance (16 calls × 32 individuals × 5 distances = 2560 calls). A detailed analysis of the effect of propagation on the acoustical structure of these calls can be found in Mouterde et al. (2014b). For the neural recordings, the naturally propagated calls were cut and centered in a 436-ms-long window to accommodate the longest recorded call. The calls were thus presented over a background of natural noise that was present throughout the whole stimuli (i.e., also before the onset and after the offset of the calls, within this 436 ms window). The calls had a mean (±SD) duration of 240 ±57 ms for female vocalizers and 149 ± 52 ms for male vocalizers, and a mean (± SD) fundamental frequency of 549 ± 126 Hz for female vocalizers and 803 ± 164 Hz for male vocalizers. All these signals were high-pass filtered with a cutoff frequency of 500 Hz. This frequency cutoff is below the lower frequency threshold of the zebra finch's audiogram (Okanoya and Dooling, 1987), and was thus used to obtain SNR values of biological significance. We calculated the SNR of our stimuli by estimating the power of the noise before and after the onset and offset of the signal and comparing with the power of the signal + noise found at the center of the window. The power was calculated by taking the time average of the square of the high-pass filtered sound pressure waveform. Using *P* for power, the subscript *C* for the estimates performed in the center of the window where both the call and noise are present, and the subscript *B*&*F* for estimates performed before and after the onset of the call, the equation for *SNR* can be written as follows:

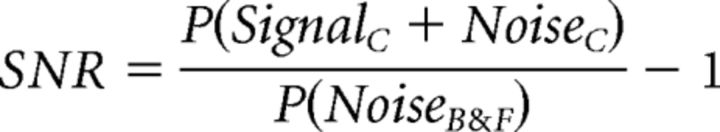
 This equation can be used because the signal and the noise are uncorrelated; therefore, *P*(*Signal_C_* + *Noise_C_*) = *P*(*Signal_C_*) + *P*(*Noise_C_*). In addition, the noise is stationary on this short time scale; therefore, *P*(*Noise_C_*) ≈ *P*(*Noise_B_*_&_*_F_*) ≈ *P*(*Noise*). The *SNR* was calculated separately for all 2560 stimuli used in the experiments and averaged across individuals and calls to obtain an average value at each propagated distance.

**Figure 1.**
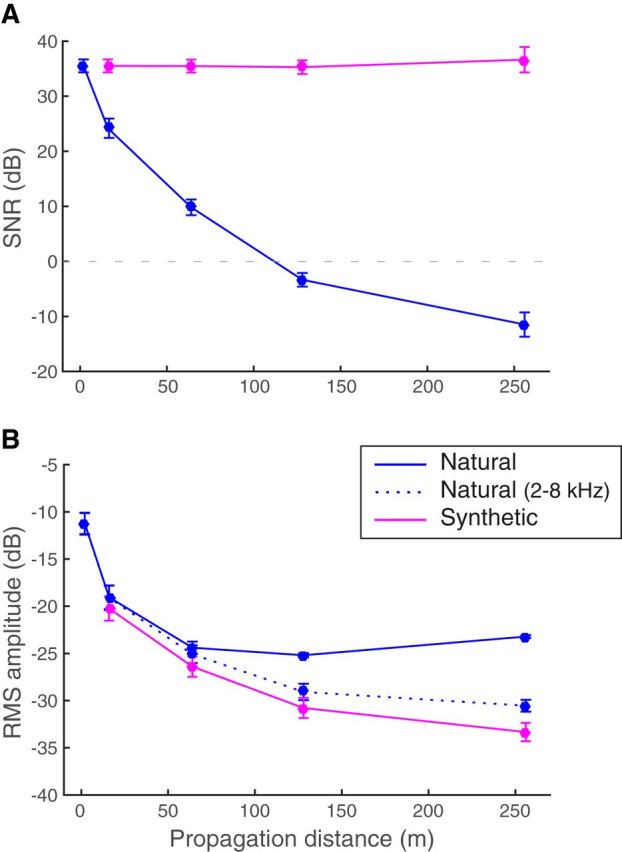
Comparison of *Natural* and *Synthetic* stimuli. ***A***, The mean *SNR* for all stimuli at each tested distance. ***B***, The mean intensity level as measured by the root mean square (RMS) amplitude. Solid lines indicate values for the stimuli broadcasted during the electrophysiology recordings (high-pass filtered above 500 Hz). Dotted line indicates the RMS values of the *Natural* stimuli filtered from 2 to 8 kHz that were used to calculate the intensity gains between distances. Error bars indicate SD. As seen in ***B***, the intensity of the *Synthetic* stimuli matched that of the *Natural* stimuli band-passed filtered from 2 to 8 kHz (dotted line). The curve representing the RMS of the *Natural* stimuli shows an elevated intensity compared with that of the *Synthetic* stimuli, in particular at the longer distances, and this is due to the fact that in *Natural* stimuli signal intensity decreases with distance while the background noise remains at approximately constant intensity at all recorded distances (at ∼−25 dB on our scale), whereas in the *Synthetic* stimuli the same background noise (recorded at 2 m) is reduced in intensity along with the signal.

To create the *Synthetic* stimuli, we used the set of *Natural* calls recorded at 2 m and reduced the gain of each call so that its amplitude matched the amplitude of the same call that had been propagated at each other distance. Gains correspond to the scaling factors that were applied on the waveforms of bandpass-filtered *Natural* calls at 2 m to match the same bandpass-filtered *Natural* calls at every other propagation distance. Indeed, to match the amplitude of the actual signal rather than the noise, the *Natural* calls used to calculate the gains were bandpass-filtered from 2 to 8 kHz so as to remove most of the background noise. The best match between time-varying amplitudes was obtained by minimizing the mean squared error. Then, *Synthetic* calls for each distance were obtained by multiplying each original *Natural* stimulus at 2 m (high-pass filtered with a cutoff frequency of 500 Hz only, as explained above) with its corresponding gain. We thus obtained *Synthetic* calls for 4 distances (16, 64, 128, and 256 m) for which the SNR was the same as the 2 m call (Fig. 1*A*), but the amplitude matched the amplitude of the *Natural* call at these 4 distances (Fig. 1*B*). The database of *Synthetic* stimuli consisted of 16 different calls per individual for the last 4 propagation distances (16 calls × 32 individuals × 4 distances = 2048 calls).

##### Animal procedures and electrophysiological recording protocol.

Eight adult zebra finches (4 males and 4 females) were used as subjects in the electrophysiology experiments. As explained in more detail by Elie and Theunissen (2015), extracellular recordings from ensemble of single units were obtained in urethane-anesthetized subjects, immobilized on a stereotaxic apparatus, using one or two 16 channel electrode arrays (Omn1010, Tucker-Davis Technologies) consisting of 2 rows of 8 electrodes (width 0.5 mm, length 1.75 mm, distance between two electrodes within one row: 250 μm). The electrode arrays were lowered into the auditory forebrain using a microdrive. Each electrode had previously been coated with DiI stain (Invitrogen) so as to facilitate the electrodes localization in the brain during the histological analysis. The recording took place in a double-walled anechoic chamber (Acoustic Systems) where a loudspeaker (Blaupunkt T-line) was used to broadcast the stimuli. The volume of the loudspeaker was set to deliver zebra finch calls at 70 dB SPL (Digital Sound Level Meter, RadioShack, weighting Type B) and was placed 20 cm in front of the subject's head. Extracellular voltages were recorded with a system from Tucker-Davis Technologies. All animal procedures were approved by the Animal Care and Use Committee at the University of California Berkeley.

We recorded multiunit responses from each electrode in the array at two to six recording depths per subject. These multiunit recordings were then spike sorted off-line as described below. Of the 8 total subjects, neural responses of 6 were recorded with a single array in one hemisphere (4 in the right hemisphere, 2 in the left hemisphere), and neural responses of 2 subjects were recorded simultaneously with two arrays, one in each hemisphere. The electrode arrays spanned the mediolateral (from 0.25 to 1.5 mm lateral from the *y*-sinus) and the rostrocaudal (from 0.25 to 2.7 mm rostral from the *y*-sinus) axes of the auditory forebrain. The depth of the recording sites spanned between 1.15 and 2.13 mm from the brain surface, and the minimum distance (depth) between two sites was 100 μm. For each electrode in the array, spike arrival times and spike waveforms (snippets) were recorded by thresholding the extracellular voltage trace during silence periods using Tucker-Davis Technologies OpenEx automatic threshold. These snippets were sorted off-line to extract responses from single units (see below).

For each recording site, calls from 4 or 8 different vocalizers from the same sex were broadcasted to the subject; sex was swapped from one site to the other in the same subject. The call identities selected for each site (i.e., vocalizer names and call renditions) were selected randomly from the database; and for each call identity, the calls recorded at all 5 distances were used for the *Natural* stimuli, and (depending on the protocol type; see below) the synthetic calls for all 4 distances were used for the *Synthetic* stimuli. To limit the recording time for each site, the number of calls selected per vocalizer varied for each condition: two subjects were only tested with *Natural* stimuli (Nat-only protocol) and heard 8 different renditions of the calls of 8 vocalizers at each site (i.e., a total number of 8 vocalizers × 8 calls × 5 Nat distances = 320 different calls per site); for the other six subjects, tested with both *Natural* and *Synthetic* stimuli (Nat+Syn protocol), some were tested with 4 vocalizers and heard 8 different renditions per vocalizer, whereas others were tested with 8 vocalizers heard 4 different renditions per vocalizer (i.e., a total number of 4 or 8 vocalizers × 8 or 4 renditions × 5 Nat distances + 4 or 8 vocalizers × 8 or 4 renditions × 4 Syn distances = 288 different calls per site). The interstimulus interval was uniformly distributed between 1 and 3 s to prevent any rhythmic pattern that could potentially entrain the neurons or generate expectations. We repeated the presentation of all the stimuli 8 times; and for each of these 8 trials, the order of stimuli presentation was randomized. In this manner, we avoided any stimulus-dependent adaptation.

##### Histology and anatomical localization of electrodes.

After the recording, the bird was terminally anesthetized with an overdose of isoflurane and transcardially perfused with Phosphate Buffered Saline (PBS) (Sigma Chemical), followed by 4% formaldehyde. In preparation of the histological procedures, the brain was sunk in 4% formaldehyde followed by 30% sucrose, before being frozen using liquid nitrogen. The brain was then sliced frontally or parasagitally in 20-μm-thick sections using a freezing microtome. Alternating brain sections were stained with either cresyl violet or DAPI nucleic acid stain and were used to localize electrode tracks (with the DiI stain marking each electrode emplacement) and histological regions. These observations were made using a Zeiss AxioImager M2 fluorescence microscope fitted with a camera (Retiga 1350 EX, QImaging).

Because of unreliable DiI stain markings, we were unable to localize the electrodes from 2 subjects of the 8 tested. Localization of the electrodes for the 6 remaining subjects involved measuring the distance from the entry point of the electrodes in the brain to their deepest point and comparing it with the depth of the last recording site as shown on the microdrive used during the recording; recording site localization could then be achieved from the coordinates of each site obtained from the microdrive. Using well-known landmarks, such as the lamina mesopallialis (known in the old nomenclature as hyperstrial lamina) and the dorsal lamina pallio-subpallialis (previously called the medullary lamina), and differences in cell density as described in the literature (Fortune and Margoliash, 1992), recording sites were then assigned to either caudal mesopallium (CM) (lateral: CLM; or medial: CMM), caudal medial nidopallium (NCM), or to the following subregions or group of subregions of the Field L complex: L1, L2, and L3/L. We chose to categorize our localization data for Field L into these three categories only (not subdividing L2 in L2a and L2b, and grouping L3 and L), as we found that subdividing further would require taking too many assumptions and would thus jeopardize the scientific validity of our results. Following guidelines found in the literature (Vates et al., 1996), we chose to approximate the limit between CMM and CLM as being 800 μm away from the midline.

To compare histological localization of electrodes between subjects, we used the depth of the recording sites and the rostrocaudal distance of each electrode to the *y*-sinus as coordinates in a 2D sagittal representation of the electrode sites. Of the 6 subjects for which histological information was available, one was recorded in both hemispheres at the same time; as the penetration angle of the electrode array was different for the left hemisphere in this dual electrode recording compared with all other penetrations, data recorded in this hemisphere were not included in the 2D representations. As a result, 521 units were used in these 2D representations out of 1322 total. Furthermore, the penetration point of the electrodes on the mediolateral axis could vary slightly from one animal to another, making direct comparisons of each row of electrodes for all subjects inaccurate. Therefore, we measured the distance of each electrode row to the midline for all birds, and three distinct groups clearly appeared on the distribution of these distances; we thus grouped electrode rows from different birds in either of these groups, depending on their distance to the midline (see Fig. 13*D*): medial (0.3–0.6 mm), middle (0.7–1.1 mm), or lateral (1.2–1.5 mm).

##### Data processing and analysis: selection of single auditory units.

We recorded multiunit activity in 640 locations using the protocol described above. These multiunits were sorted into single units based on the spike shapes, and sorted again on the basis of the quality of their responses to sounds (Elie and Theunissen, 2015). This process yielded 1322 single auditory units, and all subsequent analyses were done on this dataset of single units. All units were tested with the *Natural* stimuli (Nat-only and Nat+Syn protocols), and 1083 units were tested with the *Synthetic* stimuli (Nat+Syn protocol).

Briefly, the spike sorting was performed using a semiautomatic custom made program written in MATLAB (The MathWorks, RRID:SCR_001622) that used both unsupervised (*k*-means) and supervised clustering algorithms (Random Forest). In a first stage, we manually chose templates for single spike shapes by means of a GUI and exploratory cluster analysis using *k*-means algorithm. In a second stage, these templates were used to train a Random Forest, which was then used to classify the remainder of the spikes into single units, noise, or nonclassifiable units (multiunits). To further identify single units among spike-sorted units, the quality of the spike sorting was assessed both visually by superposing all spike snippets of each unit and quantitatively by calculating a measure of the variability within spike shapes and comparing it with the same measures obtained from a selection of units that could be very clearly identified as single units because of their large amplitude and unique shape (for more details on the spike sorting protocol, see Elie and Theunissen, 2015).

As experimenters, we could easily further classify single units in two groups: narrower spikes with more symmetric positive and negative peaks and wider spikes with more asymmetric positive and negative deviations. To classify the single units systematically, we calculated the maximum slope of the raising phase of the snippets. The distribution of these slopes was clearly bimodal (data not shown), and a threshold for classification was used in the trough between the two peaks. A very similar grouping was also obtained by performing a *k*-means unsupervised clustering in the space of the Principal Component Analysis performed on the mean spike shape. This classification led to 783 narrow spikes and 539 broad spikes (59% and 41%, respectively). The narrow spikes had a width at half-maximum of 0.24 ± 0.003 ms (SE), whereas the broad spikes had a width at half-maximum of 0.27 ± 0.002 (SE). The narrow spikes had a stimulus-driven rate estimated from the response to distance calls at 2 m of 19 spikes/s, whereas the mean rate of the broad spike neurons was 12 spikes/s (*t*_(1320)_ = 7.36, *p* < 10^−4^). Similar grouping of single units in the avian auditory pallium based on spike shape has been observed by other groups (Nagel and Doupe, 2008; Meliza and Margoliash, 2012; Schneider and Woolley, 2013; Yanagihara and Yazaki-Sugiyama, 2016).

Finally, we selected units that were responsive to acoustic stimuli (i.e., auditory units). A unit was defined as auditory if it had reproducible spike patterns in response to the same stimuli. To quantify “reproducible spike patterns,” we estimated the coherence between the average response from one half of the trials to the average response of the other half of the trials (Hsu et al., 2004a). This coherence was bias corrected, and SE estimates were obtained using jackknife procedure and multitapered estimates of the cross and self-spectra (Efron, 1982). Furthermore, we used similar calculations on neural responses to silence, to obtain an upper bound on values that could be obtained by chance even after bias correction. A unit was considered auditory if its bias corrected coherence value was above this upper bound.

##### Characterization of the neural responses.

As explained above, the stimuli were composed of distance calls centered in a 436 ms window. Thus, these stimuli included short sections of natural background noise before the onset and offset of the stimulus. The analysis for characterizing the neural responses (spike rate and spike patterns) was performed in this same 436 ms window. We quantified the neural discriminability of single units for calls from different vocalizers and propagated at different distances using an optimal decoder procedure of complete spike patterns (Wang et al., 2007; Gastpar et al., 2010; Amin et al., 2013; Elie and Theunissen, 2015). In brief, each of the 8 spike trains obtained for each stimulus was compared, by calculating van Rossum distances (see below), to average response templates obtained for all the stimuli. The average response templates were updated at each new tested spike train and were obtained for each stimulus using a random selection of 7 spike trains of the 8 trials or using the remaining 7 spike trains in the case of the stimulus from which the tested spike train was drawn. The stimulus template yielding the smallest distance designated the stimulus that was decoded. This process yielded a confusion matrix showing the joint probabilities of the predicted versus actual responses (i.e., the probabilities of classifying each spike train as belonging to its corresponding stimulus or to another stimulus). From these probabilities, it is possible to estimate the “information content” of neural responses by calculating the mutual information (*MI*) between predicted stimuli and actual stimuli as follows:

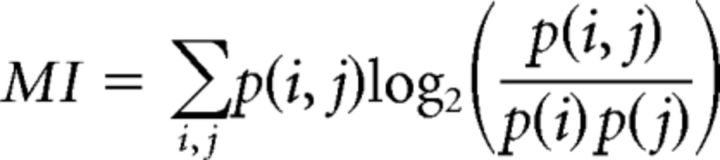
 Here the probability of the actual stimulus, *p*(*i*), depends on the number of spike trains obtained for each vocalization while *p*(*i*, *j*) and *p*(*j*) are obtained from the confusion matrix and Bayes' theorem, respectively. The mutual information of a uniformly distributed confusion matrix tends toward zero, whereas the mutual information of a highly organized matrix (e.g., if the highest probabilities are found in the diagonal) tends toward its maximum: the log_2_ of the number of categories (e.g., 2 bits for a matrix comparing 4 individuals and 3 bits for 8 individuals).

The van Rossum distance (van Rossum, 2001) corresponds to a measure of spike train similarity calculated as the Euclidian distance obtained between spike trains convolved with decaying exponentials. Depending on the time constant of the decaying exponential, the van Rossum distance explores a neural code that ranges from low to high temporal resolution. At one extreme, van Rossum distances obtained with very large time constants are very similar to distances based on the absolute value of the difference in mean firing rate. For illustrative purposes (see Figs. 5, 6), we also estimated confusion matrices and MI values with these absolute mean rate differences. At the other extreme, the van Rossum distance calculated using exponentials with very short time constants captures fine temporal structure in spike patterns that potentially code stimulus information but could also correspond to neural variability. Here we calculated van Rossum distances with 7 time scales (1, 3, 5, 10, 30, 50, and 100 ms) and obtained confusion matrices and corresponding MI values for each time scale.

**Figure 2.**
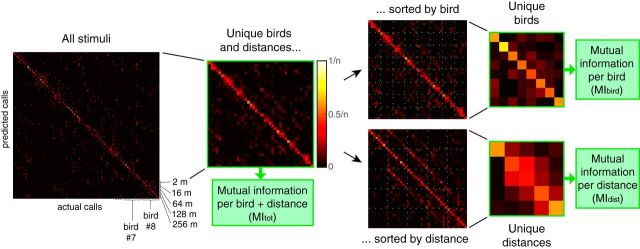
Categorization of data and measure of discrimination performance. For each unit, we obtained a global confusion matrix from the calculation of all pairwise spike train distances for all stimuli (matrix on the left of the figure). The stimuli vary along dimensions of vocalizer, distance, and call rendition. This matrix, containing the joint probabilities of the predicted versus actual stimuli, is organized first per vocalizer (8 total in this example); within each vocalizer, all 5 distances are represented; and within each distance, all particular call instances are exhibited (4 total in this case). We collapsed this matrix into a smaller matrix containing information about individuals and distances only (i.e., joint probabilities for the classification of particular renditions within each individual + distance category were added to obtain one single value for this category). From this reduced matrix, we calculated the mutual information pertaining to the discrimination of individuals and distances, referred to as *MI_tot_*. We then collapsed this matrix further, either per individuals or per distances, to obtain the mutual information pertaining to the discrimination of individuals only (*MI_bird_*) or distances only (*MI_dist_*). The colorbar for the values of joint probabilities is scaled between 0 and the maximum possible value (1/*n*) for each matrix, *n* being the number of lines in the matrix.

We first calculated for each unit a set of original confusion matrices containing the information about all *Natural* stimuli (individuals, distances, and particular call renditions; Fig. 2). We then collapsed each matrix into a smaller matrix containing information about vocalizers and distances only (i.e., joint probabilities for the classification of all call renditions within each vocalizer + distance category were added to obtain one single value for this category). From this reduced matrix, we calculated the mutual information pertaining to the discrimination of vocalizers and distances, referred to as *MI_tot_* (Fig. 2) (for additional details, see Elie and Theunissen, 2015). We then collapsed this matrix further, either per unique vocalizers or per unique distances, to obtain the mutual information pertaining to the discrimination of vocalizers regardless of distances, *MI_bird_*, or of distances regardless of vocalizers, *MI_dist_*. For each of the three mutual information calculations, we chose the time constant yielding the highest value of mutual information and used this value for further analysis. In that sense, every single value of mutual information for each unit reflected the information content of this unit using the best possible time code.

As we wanted to further investigate the impact of increasing propagation distances on the discrimination of individual identity, we calculated new confusion matrices separately for each distance, and collapsed probabilities of classification of call renditions within each matrix as explained above by grouping all calls produced by the same individual. We calculated from these matrices the mutual information pertaining to the discrimination of vocalizers at each distance, referred to as *MI_bird/dist_*. In this case, as we needed to compare directly the MI values between distances within the same unit, we had to decide on a similar time scale: we needed to apply the same time constant among the 7 tested to calculate comparable mutual information at all distances. This optimal time scale was chosen as the time constant that yielded the best discrimination performance at 2 m with *Natural* stimuli.

We performed the same calculations using the *Synthetic* stimuli in place of the *Natural* stimuli for distances greater than 2 m. All values of mutual information in the text below pertain to the *Natural* stimuli unless specified being obtained from *Synthetic* stimuli.

##### Correction of mutual information values and percentage of correct classification.

Next, to compare any of the values of mutual information described above (*MI_tot_*, *MI_bird_*, *MI_dist_*, *MI_bird/dist_* at all distances, for *Natural* and *Synthetic* stimuli), between cells that were exposed to different numbers of categories (e.g., 4 or 8 different vocalizers, or 5 distances), and to eliminate any positive bias in the evaluation of discrimination performance, we calculated a corrected value, *MI_c_*, for each value of mutual information, *MI_Obs_*, as follows:

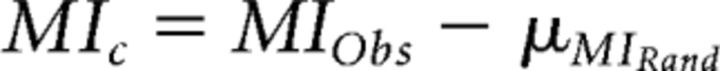
 where μ*_MI_Rand__* corresponded to the mean of the mutual information values obtained using a bootstrapping resampling technique (described below) on the confusion matrix used to calculate *MI_Obs_*. All the values of mutual information presented in Results and figures are corrected values (*MI_c_*).

The bootstrapping resampling technique was performed as follows: for each unit, we obtained 1000 bootstrap versions of the original confusion matrix containing information about all *Natural* stimuli, and of the original confusion matrices calculated per distance, by randomizing the assignment of the predicted stimuli. Each of these “scrambled” confusion matrices was then collapsed so that we could calculate the various values of mutual information: *MI_tot_*, *MI_bird_*, *MI_dist_*, and *MI_bird/dist_*. This process yielded for each original confusion matrix a distribution of values of mutual information that could be obtained by chance (*MI_Rand_*). Finally, to obtain values of MI in bits per second, the information values were simply divided by the duration of the analysis window (0.436 s).

To provide more intuitive values for discrimination performance, we also calculated the percentage of correct classification above chance for each confusion matrix, by adding the joint probabilities of the diagonal of the matrix (which is exactly equal to taking the average of the conditional probabilities in this symmetric dataset), and subtracting the chance level. For *MI_bird_* and *MI_bird/dist_*, the chance level was 25% for subjects tested with 4 vocalizers and 12.5% for subjects tested with 8 vocalizers, whereas for *MI_dist_* the chance level was always 20% as 5 distances were tested. This calculation provided a “corrected” percentage value, enabling us to compare subjects tested with a different number of individuals.

##### Investigation of the invariance quality of the vocalizer discrimination to sound degradation.

To characterize the effect of propagation distance on the discrimination of the identity of the vocalizer for each unit, we fitted an exponential model to the (corrected) *MI_bird/dist_* (for each type of stimuli, *Natural* and *Synthetic* calls) with distance being a predictor. These models yielded a measure of the discrimination decay length for each type of stimuli, that is, the distance (in meters) over which the unit loses one bit of mutual information. The exponential model is as follows:

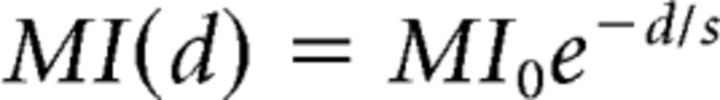
 where *d* is the propagation distance, *MI*_0_ is a constant, and *S* the discrimination decay length, in meters. For this fit, we only used distances up to the first (and not including) distance where *MI_bird/dist_* was negative or null (or all the distances if all *MI_bird/dist_* > 0). In the case when none or only one *MI_bird/dist_* value was available, no discrimination length value could be retrieved (this concerned 303 units of 1322 total for the *Natural* stimuli and 498 units of 1083 total for the *Synthetic* stimuli).

##### Investigation of the selectivity of responses for vocalizers and for distances.

To investigate the selectivity for particular distances or vocalizers, we computed a measure of selectivity based on the entropy of the distribution of conditional probabilities in the diagonal of the confusion matrices, the Global Selectivity (*GS*) (Elie and Theunissen, 2015). *GS* is defined as follows:

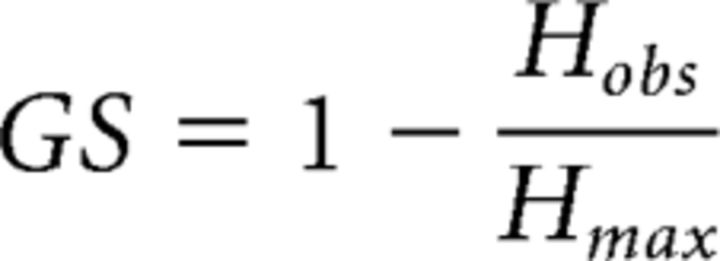
 with *H_obs_* the observed entropy based on the normalized probabilities in the diagonal of the confusion matrix *p*(*i, i*) and *H_max_* the maximum possible entropy obtained if these probabilities had been equal. These entropies are calculated as follows for a matrix of size *N*:

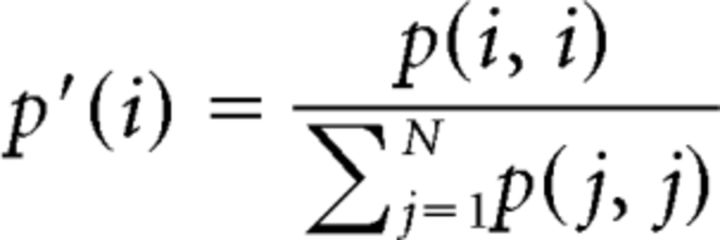


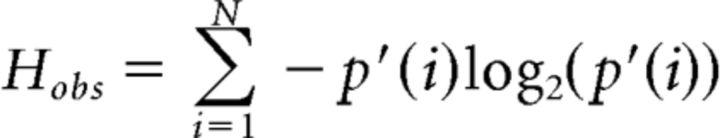


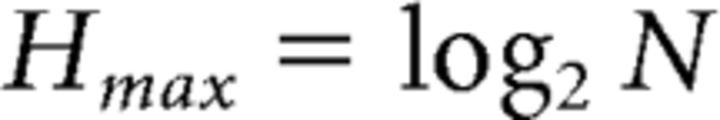
 The entropy *H_obs_* measures the degree of similarity in the probabilities found in the diagonal of the confusion matrix: very similar values (i.e., exhibiting a complete absence of selectivity at the unit level) would yield an entropy value close to *H_max_* and a *GS* close to 0, whereas a nonuniform distribution of these diagonal probability values (i.e., indicative of higher selectivity for the acoustic characteristics of certain calls) would yield a lower *H_obs_* and thus higher *GS* with a maximum possible value of 1. For each unit, *GS* was calculated on the confusion matrix obtained for discriminating distances only or vocalizers only. The observed values of *GS* were compared with the *GS* values obtained for random neuron-like confusion matrices of the same size that yielded a range of values of mutual information similar to the range obtained in the neural data. The highest probability in each line of these random neuron-like confusion matrices was placed along the diagonal (to generate information corresponding to discrimination and not systematic errors). As it was the case for the neural data, the value of mutual information of each random neuron-like matrix was bias corrected by subtracting the mutual information obtained from totally random matrices with the same value of *GS*.

##### Generation of functional maps and statistical analyses.

We combined the anatomical location of each recording site (measured as the exact rostral/caudal distance from the *y*-sinus, the exact depth from the brain surface and as a medial (0.3–0.6 mm), middle (0.7–1.1 mm), or lateral (1.2–1.5 mm) position; see Histology and anatomical localization of electrodes) with the MI measures (*MI_bird_* and *MI_dist_*) to obtain functional maps. To create these functional maps, we first averaged the mutual information values that occurred at the exact same depth and rostral/caudal positions in each of the three medial/lateral positions (from single units recorded on the same electrode site) and then performed 2 d linear interpolation (using the MATLAB function *griddata*) to obtain information values at equally spaced grid points spanning the region of interest. To assess whether the functional maps exhibited correlated spatial patterns, we calculated the correlation coefficient between *MI_bird_* and *MI_dist_* across pair values taken at the grid points. To assess these correlations along cardinal dimensions (here rostral/caudal vs dorsal/ventral or depth), we simply averaged these grid values along each axis. Finally, to assess the significance of the correlations, we generated random maps by bootstrapping. In the bootstrap, the average values of mutual information obtained for each recording site were randomly permuted (i.e., assigned randomly to one of the recording sites). This randomization was performed separately for *MI_bird_* and *MI_dist_*. Permutations were repeated 1000 times and correlation coefficients were obtained for each of the random maps. The actual correlation values were then compared with the distribution of bootstrapped correlation values to obtain a *p* value.

## Results

### Identifying the neural substrate for the discrimination of individual signatures and propagation distances

**Figure 3.**
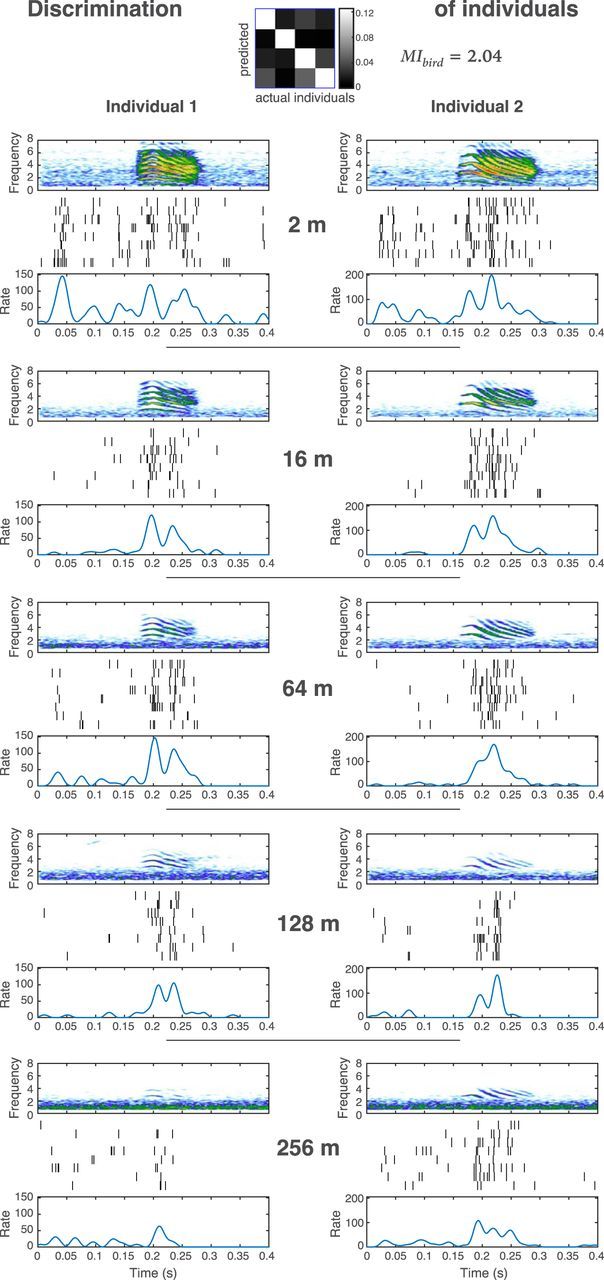
Comparison of the responses of a unit highly discriminative for the signature of individual vocalizers to the call of two different males, at all tested distances. For each propagation distance, the spectrogram of the stimulus call is shown on top, followed by the spike trains for the 8 presentations, and by the peristimulus time histogram averaging these 8 presentations. Sound frequency is given in kilohertz and rate in spikes per second. The confusion matrix shows the discrimination ability between individuals (taking into account all distances): the whiter the diagonal, the higher the individual discrimination. The mutual information value calculated from this matrix (*MI_bird_*) is given in bits per second. In this particular example, the neuron also responds to background sounds. This is clearly observed for a noise preceding the call of Individual 1 at 2 m.

**Figure 4.**
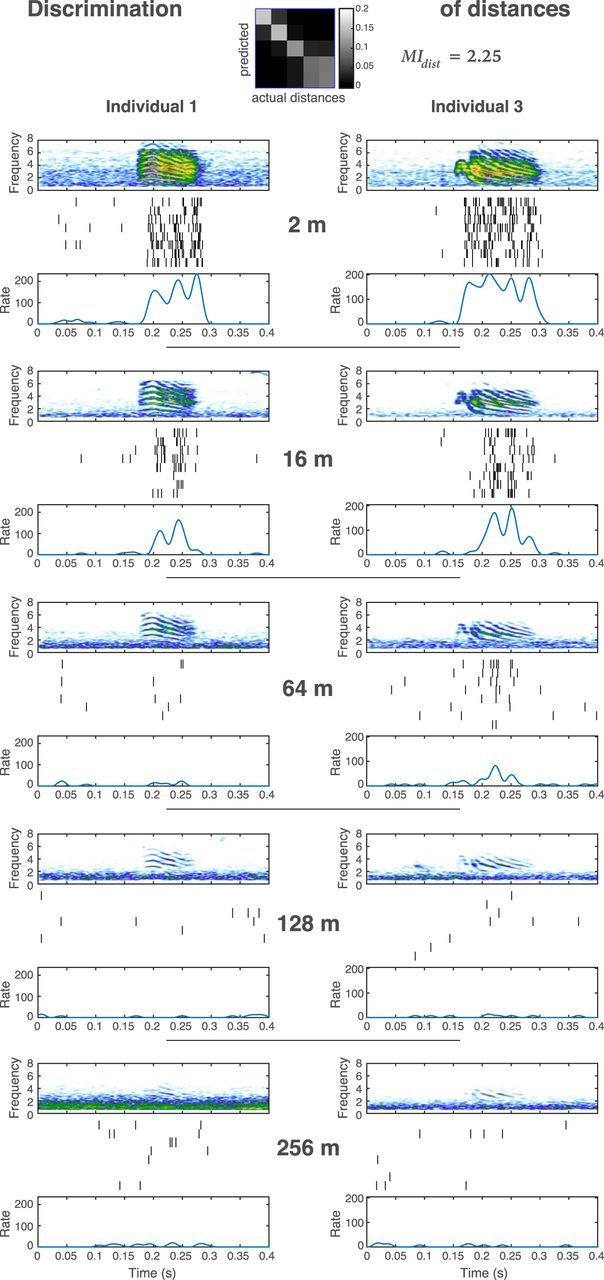
Comparison of the responses of a unit highly discriminative for the distances to the call of two different males, at all tested distances. The stimulus on the left column corresponds to a different rendition of a call from the same individual as that of Figure 3 (Individual 1). For each propagation distance, the spectrogram of the stimulus call is shown on top, followed by the spike trains for the 8 presentations, and by the peristimulus time histogram averaging these 8 presentations. Sound frequency is given in kilohertz and rate in spikes per second. The confusion matrix shows the discrimination ability between distances (taking into account all individuals): the whiter the diagonal, the higher the discrimination. The mutual information value calculated from this matrix (*MI_dist_*) is given in bits per second. Compared with the unit of Figure 3, this unit is more sensitive to changes in stimulus intensity with higher rates at 2 m and lower rates at 64, 128, and 256 m.

We found single neurons showing high discrimination for the vocalizer identity at short, as well as at long distances: these neurons thus keep their ability to discriminate the vocalizer's identity despite propagation-induced sound degradation. We also found neurons showing high discrimination for propagation distance (i.e., an ability to code information about the distance separating the emitter and the receiver birds). We illustrate both types of responses in Figures 3 and 4 where we compare the responses of two units to the stimulus call from two different individuals, at all tested distances. In Figure 3, we show example spike rasters obtained in response to two different males' calls in a unit with a high performance for vocalizer discrimination regardless of distance (i.e., high value of *MI_bird_*, and high probability values in the diagonal of the “per bird” confusion matrix). In this example, the firing rate during the stimulus is clearly higher than before or after the stimulus for both individuals at all distances, and the spike patterns are different between the two individuals, illustrating the fact that this unit codes different individuals differently and preserves some aspects of that differential code across all distances. In Figure 4, we show example spike rasters of a different unit with a high performance for the discrimination of distances regardless of the emitter's identity (i.e., high value of *MI_dist_*, and high probability values in the diagonal of the “per distance” confusion matrix), to two calls of different males (the stimulus on the left column is from the same vocalizer as the one on the left column in Fig. 3: Individual 1). In this example, the firing rate during the stimulus is higher than before or after the stimulus for both individuals from 2 to 64 m, but not at further distances. At 128 and 256 m, no specific spike pattern is discernable regarding a response to the stimulus for both individuals. However, the constant decrease in spike rate from short to long distances illustrates the fact that this unit codes propagation distance.

**Figure 5.**
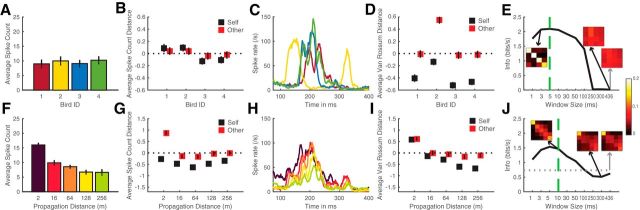
Comparison of the discrimination performances obtained using spike counts versus spike patterns for a unit that is highly discriminative for the signature of individual vocalizers (same unit as in Fig. 3). Top row, Investigation of the unit's discrimination of vocalizers. Bottom row, Investigation of the unit's discrimination of the distances. ***A***, ***F***, Average spike count obtained for the stimuli emitted by each vocalizer (***A***), and at each propagation distance (***F***) during the 436 ms analysis window. ***B***, ***G***, Average pairwise distance between neural responses calculated as the absolute value difference in spike count. Black squares represent the average distance between the neural responses obtained (***B***) with different stimuli from the same vocalizer or (***G***) with different stimuli propagated at the same distance (Self distance). Red squares represent the distance between neural responses obtained with the stimuli from each vocalizer (***B***) or each distance (***G***) with the stimuli from all other vocalizers (***B***) or distances (***G***) (Other distance). Average distances are *z*-scored to compare distances obtained with rates and patterns. For a given distance or individual, the discrimination is maximal when the Self distance is higher than the Other distance (red squares above black squares). ***C***, ***H***, Average spike patterns centered around the stimuli (75–400 ms) obtained for stimuli emitted by each vocalizer (***C***), and at each propagation distance (***H***). Color code is identical between ***A*** and ***C***, and between ***F*** and ***H***. ***D***, ***I***, Average distance between neural responses calculated as the van Rossum distances between spike patterns (see Materials and Methods). Same as in ***B***, ***G***, distances are *z*-scored and the discrimination performance correlates with the positive difference between red and black squares. ***E***, ***J***, Mutual information about the vocalizer identity (***E***) and the propagation distance (***J***) calculated from confusion matrices based on the van Rossum distances between spike patterns analyzed at various time scales (window sizes; see Materials and Methods). Green vertical line on each graph indicates the scale at which the spike patterns shown in ***C*** and ***H*** were analyzed to obtain maximal values of information. Gray dotted horizontal line (close to zero in ***E***) indicates the mutual information achieved with neural distances based solely on spike counts (rate code). The confusion matrices used to calculate the mutual information pertaining to vocalizer identity (***E***) and propagation distance (***J***) are shown as insets in ***E***, ***J***. Black arrows indicate confusion matrices corresponding to the best and the worst time scale for the analysis with the van Rossum distances. Gray arrow indicates confusion matrix obtained with the spike count distances. For this unit, which belongs to the bird-specific cluster, using a measure of distance that investigates the temporal code (van Rossum distance, as opposed to spike count) greatly enhances our ability to detect the neural discrimination performance for vocalizer identity (compare ***B***, ***D***; see values of mutual information achieved in ***E***) and, to a lesser extent, for propagation distance.

**Figure 6.**
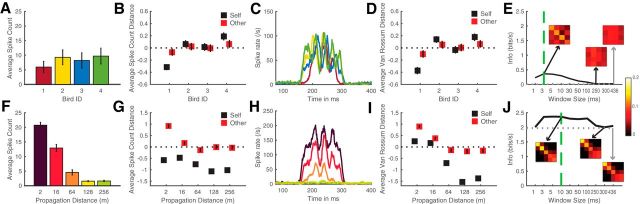
Comparison of the discrimination performances obtained using spike counts versus spike patterns for a unit highly discriminative for the distance of propagation (same unit as in Fig. 4). Detailed descriptions of each panel are the same as Figure 5. For this unit, using a measure of distance that investigates the temporal code (van Rossum distance, as opposed to spike count) only slightly enhances our ability to detect the neural discrimination performance for vocalizer identity or propagation distance.

The observations we made regarding the response of our illustrative neurons in Figures 3 and 4 can be quantified by calculating how vocalizer identification or distance can be discriminated from the neural response when it is described in terms of spike rates or spike patterns. We performed such calculations for our entire dataset but show the details of such calculations for our two example neurons first. Figures 5 and 6 show the average spike counts (Figs. 5*A*, 5*F*, 6*A*, 6*F*) and patterns (Figs. 5*C*, 5*H*, 6*C*, 6*H*) that are obtained for 4 individual calls regardless of rendition or distance (Figs. 5*A*, 5*C*, 6*A*, 6*C*) and for distances regardless of individual or renditions (Figs. 5*F*, 5*H*, 6*F*, 6*H*), calculated for the example neurons shown in Figures 3 and 4, respectively. The neuron shown in Figures 3 and 5 has similar spike counts (Fig. 5*A*,*B*) but different spike patterns (Fig. 5*C*,*D*) for calls emitted by different birds. These different spike patterns yield high discrimination and information (*MI_bird_*) when the temporal code is considered (Fig. 5*D*,*E*) but low discrimination and information when the rate code is considered (Fig. 5*B*,*E*). In contrast to the discrimination for identity, the discrimination for distance can be obtained by a rate code: the average count decreases with distance (Fig. 5*F*). A temporal code can only slightly boost the discrimination and information (*MI_dist_*) obtained from rate (Fig. 5*G*,*I*,*J*). Thus, this unit codes vocalizer identification with different spike patterns for each vocalizer that are relatively robust to distance. Besides, this unit is still able to code distance by changes in the overall rate.

In contrast to the neuron in Figures 3 and 5, the neuron shown in Figures 4 and 6 has both similar firing rates and similar firing patterns to calls emitted from all 4 individuals (Fig. 6*A*,*C*) yielding small values of discrimination and information for all integration windows (Fig. 6*B*,*D*,*E*). That same neuron, however, exhibits a firing rate that is very sensitive to distance and drops drastically from 2 to 128 m (Fig. 6*F*). The decrease in rate is observed at all time points (Fig. 6*H*). Considering a temporal code is barely boosting the discrimination of distances (Fig. 6*G*,*I*). Thus, this unit codes increasing distance with decreasing spike rate (Fig. 6*G*,*J*) and does not code individual signature. This rate coding scheme for distance is characteristic of the population (as described below and as shown in Fig. 10*C*,*D*).

**Figure 7.**
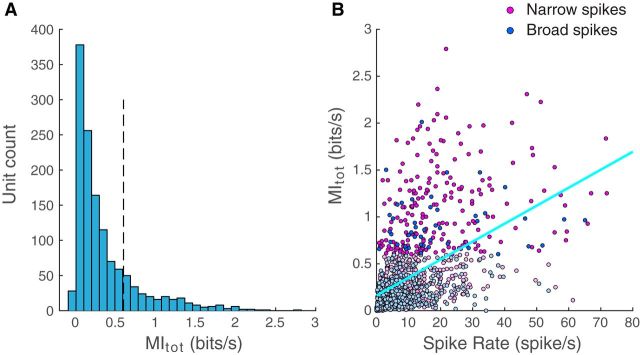
Distribution of *MI_tot_* values and correlations with spike rate. ***A***, Histogram of total information values obtained for all auditory units (*n* = 1322). Dotted vertical black line at 0.6 bit/s indicates the threshold value we chose to define highly discriminative units, the units that are found in the tail of this distribution. ***B***, Scatter plot of *MI_tot_* versus spike rate. Information is correlated with spike rate (*r* = 0.47; *p* < 10^−6^). Light blue line indicates the linear regression line. Each point corresponds to one unit and has been colored according to its spike shape. Light colors represent units with *MI_tot_* values below the 0.6 bits/s threshold. The correlation between information and rate is similar for units with broad and narrow spikes (see Results).

To analyze the range of measured discrimination of individual signatures and propagation distances for our entire population of auditory neurons, we first examined the distribution of *MI_tot_*, *MI_dist_*, and *MI_bird_* and their relationship with spike rate and spike shape. The histogram in Figure 7*A* shows the distribution of *MI_tot_* for the entire population of auditory neurons. This distribution has a strong positive skew with a long tail of single units that have high information values. We chose to examine the properties of these high information cells separately by using a threshold of 0.6 bits/s. Of 1322 auditory neurons, 245 (or 19%) had an *MI_tot_* value above this threshold. We call these neurons highly discriminative neurons. Among these 245 highly discriminative cells, 198 (81%) had narrow spikes and 47 (19%) had broad spikes. We note that previous studies had found that broad spiking neurons were more selective than narrow spiking neurons when stimulated with songs from different individuals (Nagel and Doupe, 2008; Meliza and Margoliash, 2012; Schneider and Woolley, 2013; Yanagihara and Yazaki-Sugiyama, 2016). This apparent discrepancy is addressed in the discussion and can be understood when the relationship between spike rate, spike patterns, information, discrimination, and selectivity are considered together. This proportion of narrow spikes is significantly higher than the one found for the entire population of auditory neurons recorded here (81% vs 59%; *z* test for proportion: *z* = 6.88, *p* < 10^−6^). Next, we examined the relationship between spike rate and information (Fig. 7*B*): total information is positively correlated with spike rate (*F* test: *b* = 0.019 bits/spike; *R*^2^ = 0.22; *F*_(1,1320)_ = 493; *p* < 10^−6^). Similar results are obtained when the population is divided into narrow (*b* = 0.02 bits/spike; *R*^2^ = 0.27; *F*_(1,781)_ = 294; *p* < 10^−6^) and broad neurons (*b* = 0.013 bits/spike; *R*^2^ = 0.22; *F*_(1,537)_ = 150; *p* < 10^−6^). These values are contrasted by the higher ceiling values of mutual information and stronger relationship with rate that were found in the same system when investigating the coding of the stimulus' spectrotemporal features, with averages of 7 bits/s and 0.9 bits/spike (Hsu et al., 2004b, their Fig. 5); thus, not surprisingly, these auditory neurons are also encoding spectrotemporal acoustic features that correlate to informative attributes beyond range and caller identity (e.g., call category).

**Figure 8.**
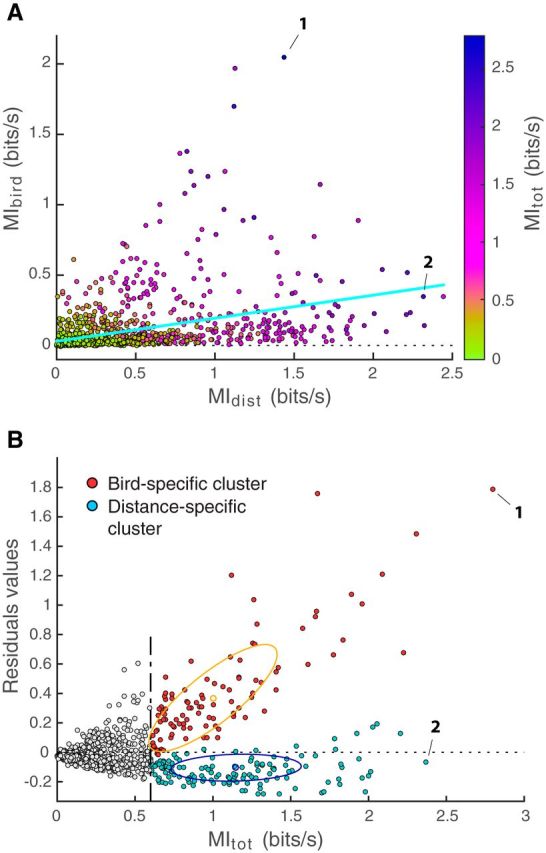
Distribution of the data and categorization of units. ***A***, All units (*n* = 1322) are plotted as a function of *MI_bird_* and *MI_dist_*. Color scale represents *MI_tot_*. Light blue represents the regression line calculated using all data points. ***B***, Residual value for each unit calculated from the linear regression shown above, as a function of *MI_tot_*. Vertical black dotted line indicates the threshold of 0.6 bits/s we used to select highly discriminative units (see Fig. 7; and Results). An unsupervised clustering based on a mixture of two Gaussians was performed on the residuals and *MI_tot_* of these highly discriminative units. This analysis defines two well-separated groups: the red cluster, with positive residual values, regroups units showing higher discrimination ability for vocalizer identity (higher *MI_bird_* values) and will be referred to as the “bird-specific cluster”; and the cyan cluster, with residual values that are negative or close to zero, regroups units showing higher discrimination ability for distances (higher *MI_dist_* values) and will be referred to as the “distance-specific cluster.” All values are shown using responses to *Natural* stimuli only. On both plots, the units used to create Figures 3 and 5, and Figures 4 and 6, are highlighted (Unit 1 used in Figs. 3 and 5; Unit 2 used in Figs. 4 and 6).

To further examine the coding properties of the highly discriminative neurons, we plotted all the units as a function of *MI_dist_* and *MI_bird_* (Fig. 8*A*). The positive slope in the cone-shaped cloud of scatter points measures the positive correlation between *MI_dist_* and *MI_bird_. MI_bird_* and *MI_dist_* are correlated in part because these component information values are correlated with *MI_tot_*, which is in turn correlated with the spike rate. However, the cone shape also suggests a form of specialization of the highly discriminative neurons with, on one end, relatively high values of *MI_dist_* and lower values of *MI_bird_*, and vice-versa on the other end. We also note that there are neurons with high values of *MI_dist_* but with close to null values of *MI_bird_*, whereas units with high *MI_bird_* all encode some distance information. We further examined this distribution by first estimating the correlation between *MI_dist_* and *MI_bird_*, shown as a regression line on the figure. As expected from the scatter, the correlation is small but highly significant (*F* test: *F*_(1,1320)_ = 246; *R*^2^ = 0.16; *p* < 10^−6^). Then, to distinguish units showing a higher discrimination for individual identity from units showing a higher discrimination for distances (compared with the mean discrimination of all units), we plotted the residuals from this linear model as a function of *MI_tot_*. As seen in Figure 8*B*, this joint distribution of residuals and information was clearly not unimodal. To capture this feature, and to methodically assign units into the two groups described above for further analysis, we fitted this distribution for the highly discriminative units (*MI_tot_* > 0.6 bits/s) using a Gaussian mixture model (*fitgmdist* function in MATLAB) with two clusters. The two Gaussians used in the fit have well-separated means and different axis in their covariance matrix (Fig. 8*B*), indicating that the observed distribution is indeed bimodal. In summary, this unsupervised clustering analysis showed two well-separated groups of highly discriminative neurons, shown in red and blue in Figure 8*B*: the red cluster, with positive residual values (mean and SD: 0.40 ± 0.36 bits/s), regrouped units showing higher discrimination ability for individuals (i.e., higher *MI_bird_* values) and will be referred to as the “bird-specific cluster”; the blue cluster, with residual values that are negative or close to zero (mean and SD: −0.12 ± 0.09 bits/s), regrouped units showing higher discrimination ability for distances (i.e., higher *MI_dist_* values) and will be referred to as the “distance-specific cluster.” A very similar grouping would have been obtained by simply dividing cells into those with positive and those with negative residuals. The unsupervised clustering approach we used showed that the distribution of data further justifies this grouping. Out of the 245 high discriminative units (*MI_tot_* > 0.6 bits/s), the bird-specific cluster contained 104 units and the distance-specific cluster contained 141 units. For the bird-specific cluster, the average *MI_bird_* was 0.55 ± 0.37 (SD) bits/s, corresponding to an average proportion of total information *MI_bird_*/*MI_tot_* of 51 ± 18% (SD), whereas for the distance-specific cluster, the average *MI_bird_* was 0.12 ± 0.10 (SD) bits/s, corresponding to an average proportion of total information *MI_bird_*/*MI_tot_* of 11 ± 6% (SD).

The proportion of broad-spike versus narrow-spike unit types in the bird-specific cluster and the distance-specific cluster were not different from the proportion of broad spike versus narrow spike in the highly discriminative units from which these two groups were derived. In the bird-specific cluster, we found 82% of narrow spikes (85 of 104) versus 81% (198 of 245) in the highly selective units (*z* test for proportions: *z* = 0.24, *p* = 0.40). In the distance-specific cluster, we found 80% of narrow spikes (113 of 141) versus 81% (198 of 245) in the highly discriminative units (*z* test for proportions: *z* = −0.2, *p* = 0.58). Therefore, there does not appear to be any relationship between putative neuronal types based on spike shape and the belonging of neurons to the distance- or bird-specific cluster. Further below, we will also analyze the anatomical locations of neurons in these two groups.

### Effect of propagation distance on the vocalizer discrimination performance: investigating the stability of the neural discrimination for vocalizer identity across propagation distances

**Figure 9.**
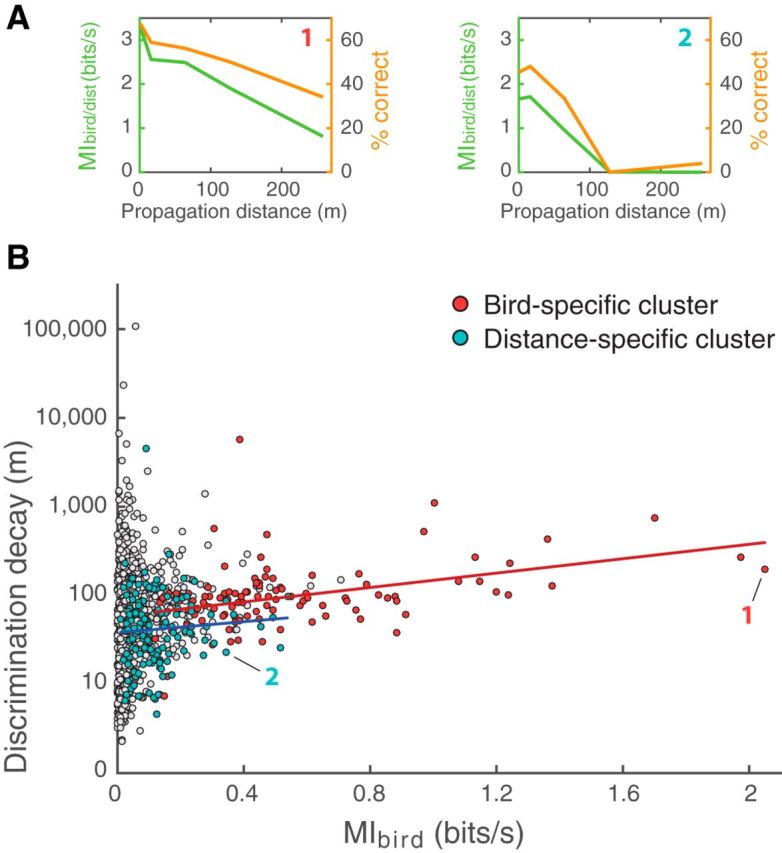
Discrimination performance of vocalizer identity and invariance to distance. ***A***, Discrimination decay curves (i.e., the discrimination performance as a function of propagation distance) are shown for the two units presented in Figures 3, 5 and Figures 4, 6, respectively: Unit 1 shows high individual discrimination ability, and Unit 2 shows high distance discrimination ability. Green represents the *MI_bird/dist_* value calculated for each distance (left *y*-axis). Orange represents the percentage of correct classification above the chance level extracted from the confusion matrices calculated for each distance (right *y*-axis). ***B***, All units for which a discrimination decay length value is available (*n* = 1019 of 1322 total) are plotted as a function of their discrimination decay length and *MI_bird_*. The discrimination decay length, shown in log axis, represents the distance (in meters) over which the unit loses one bit of mutual information about vocalizer identity. Colors represent the two clusters derived in Figure 8. The two units presented in ***A*** are highlighted. The regression lines indicate linear models fitted to the units in the bird-specific (red) and the distance-specific (blue) clusters. All values are shown using responses to *Natural* stimuli only.

To further investigate how discrimination for identity is affected by distance, we calculated the mutual information on confusion matrices computed separately for each distance, *MI_bird/dist_* (see Materials and Methods). This analysis allows us to examine the interaction between the discrimination of identity and the discrimination of distance: for example, does a bird-specific unit have very high discrimination for identity at 2 m but poor discrimination at longer distances, or is it able to maintain high levels of discrimination for identity at all distances? Figure 9*A* shows *MI_bird/dist_* and the percentage of correct classification above chance level as a function of propagation distance for the two units presented in Figures 3–6. For the first unit, which belongs to the bird-specific cluster (high discrimination for vocalizer identity), the vocalizer discrimination performance decreases slowly with distance: the probability of correct classification drops only from 67% at 2 m to 34% at 256 m. In comparison, for the second unit, which belongs to the distance-specific cluster (high discrimination for propagation distance), the mutual information about the vocalizer identity decreases rapidly, reaching zero at 128 m.

To assess the relationship between the stability of neural vocalizer discrimination across distances and the overall vocalizer discrimination performance (*MI_bird_*, which measures discrimination regardless of distance; see Materials and Methods), we represented units in terms of their discrimination decay length and *MI_bird_* (Fig. 9*B*). The discrimination decay length represents the distance (in meters) over which the unit loses one bit of mutual information about vocalizer identity. The longer the distance is, the more stable the neural discrimination is to distance. Discrimination decay length values in both clusters are significantly different, the bird-specific cluster displaying a higher discrimination length (mean decay length for bird-specific cluster: 97.6 ± 2.3 m; mean decay length for distance-specific cluster: 41.7 ± 2.6 m; two-sample *t* test: *t*_(241)_ = 7.28, *p* = 4.83 × 10^−12^).

Thus, the units displaying a higher overall performance of discrimination between vocalizers (bird-specific cluster) are those that have the longest discrimination decay length; in other words, units achieving high performance of vocalizer discrimination are the least affected by call propagation distance and obtain high discrimination by maintaining relatively high-level of discrimination across all distances.

### Effect of vocalization intensity in the propagation-induced degradation of vocalizer discrimination and relationship with spike rate

**Figure 10.**
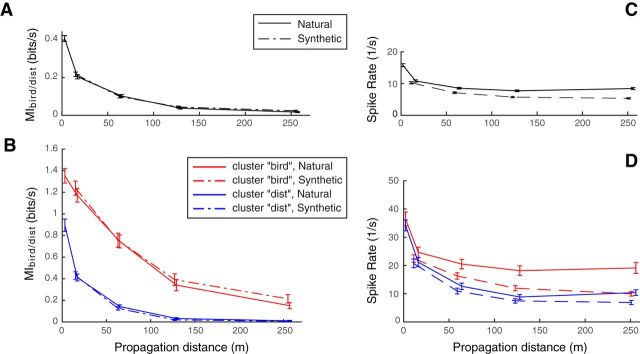
Comparison of vocalizer-identity discrimination performance for *Natural* and *Synthetic* stimuli. ***A***, ***C***, Mean decay curves for the vocalizer discrimination per distance *MI_bird/dist_* (***A***) and for the spike rate (***C***) for the *Natural* and *Synthetic* stimuli at all distances and for all units that were tested with both types of stimuli (*n* = 1083 of 1322 total). ***B***, ***D***, Same plots for units contained in the bird-specific (“bird”) and distance-specific (“dist”) clusters. Error bars indicate SEM. Small jitter along the *x*-axis (distance) has been added for better visibility.

The Nat+Syn protocol aimed at further investigating the source of the vocalizer discrimination and spike rate decays as propagation distance increases: each subject was challenged with two sets of stimuli to disentangle the overall effect of sound propagation through natural environment from the mere effect of the decrease in sound intensity on the units' responses. The *Synthetic* stimuli mimic the intensity decrease of the *Natural* stimuli at the equivalent distance (from 16 to 256 m), but with the same high SNR as the one obtained from the call recorded at 2 m (see Materials and Methods). Figure 10 shows the mean decay curves for the neural discrimination (*MI_bird/dist_*) (Fig. 10*A*) and spike rate (Fig. 10*C*) to the *Natural* and *Synthetic* stimuli at all distances and for all units that were tested with both types of stimuli (*n* = 1083 of 1322 total). On the one hand, the decay curves for *MI_bird/dist_* are strikingly equivalent and a mixed-effects linear model using the distances and the type of stimuli as categorical predictors of *MI_bird/dist_* did not reveal any significant effect of the type of stimuli or of the interaction between the predictive factors while distance was highly significant (ANOVA on the model generated with *fitlme* in MATLAB with unit as a random factor: type of stimuli, *F^Stim^*_1,8656_ = 3.61, *p^Stim^* = 0.058; distance × type of stimuli, *F*^Dist*Stim^_3,8656_ = 0.86, *p*^Dist*Stim^ = 0.46; distance, *F^Dist^*_3,8656_ = 316.9, *p^Dist^* < 10^−6^). On the other hand, the spike rate decreased more sharply to lower values with increasing distance for the *Synthetic* than for *Natural* propagated sounds (mixed effect linear model: *F^Stim^*_1,8656_ = 13.92, *p^Stim^* = 0.0002; distance × type of stimuli, *F*^Dist*Stim^_3,8656_ = 34.08, *p*^Dist*Stim^ < 10^−6^; distance, *F^Dist^*_3,8656_ = 471, *p^Dist^* < 10^−6^). The greater decrease in spike rate for the *Synthetic* sounds at long propagation distances can be explained by the fact that the *Natural* sounds have higher levels of background noise and, thus, higher overall (signal + noise) sound power (Fig. 1). Those additional noise-driven spikes, however, did not seem to affect the neural discrimination performance.

In Figure 10*B*, *D*, the mean decay curves for *MI_bird/dist_* and spike rate are plotted separately for the distance-specific cluster (retrievable decay values *n* = 132 of 141; for our exclusion criterion, see Materials and Methods) and the bird-specific cluster (*n* = 76 of 104). Just as we observed for the entire data, a mixed-effects linear model did not reveal any significant effect on *MI_bird/dist_* of the type of stimuli or of the interaction term for either of the two clusters, whereas distance was highly significant for both clusters (bird-specific cluster: *F^Stim^*_1,600_ = 2.14, *p^Stim^* = 0.14, *F*^Dist*Stim^_3,600_ = 0.32, *p*^Dist*Stim^ = 0.81, *F^Dist^*_3,600_ = 202.5, *p^Dist^* < 10^−6^; distance-specific cluster: *F^Stim^*_1,1048_ = 0.63, *p^Stim^* = 0.43, *F*^Dist*Stim^_3,1048_ = 0.48, *p*^Dist*Stim^ = 0.70, *F^Dist^*_3,1048_ = 157.1, *p^Dist^* < 10^−6^). The *MI_bird/dist_* was much greater for the bird-specific cluster than for the distance-specific cluster, but this is expected by construction. The decrease with distance of the spike rate for these two specific subsets also showed the same pattern as in the entire dataset: the rate decreased further for the *Synthetic* than for the *Natural* calls (bird-specific cluster: *F^Stim^*_1,600_ = 8.2, *p^Stim^* = 0.004, *F*^Dist*Stim^_3,600_ = 14.2, *p*^Dist*Stim^ < 10^−6^, *F^Dist^*_3,656_ = 100, *p^Dist^* < 10^−6^; distance-specific cluster: *F^Stim^*_1,1048_ = 0.46, *p^Stim^* = 0.5, *F*^Dist*Stim^_3,1048_ = 4.56, *p*^Dist*Stim^ = 0.0035, *F^Dist^*_3,1048_ = 225, *p^Dist^* < 10^−6^). Moreover, although the spike rates at 2 and 16 m are not significantly different between the distance-specific cluster and the bird-specific cluster, the rate for the distance-specific units drops fast with propagation distance, whereas the rate for the bird-specific units drops at a slower rate and remains elevated (and above the overall mean) for all propagation distances.

Therefore, it appears that, for all units, the degradation of the units' discrimination performance with distance is mostly explained by the mere decrease in intensity rather than by the increase in signal degradation or the additional spikes due to background noise. In other words, neurons appear to be very robust (i.e., almost completely invariant) to the signal degradation and to large decreases in SNR (Fig. 1); the decline in discrimination performance is a result of the decrease in intensity of the signal but not of the decrease in SNR or of propagation induced deteriorations, such as temporal smearing.

### Coding properties of neural units

Next, we examined the coding properties of the units belonging to the two clusters (the bird-specific cluster and the distance-specific cluster) by investigating the selectivity of discriminating units and the temporal resolution of the neural code.

### Selectivity

**Figure 11.**
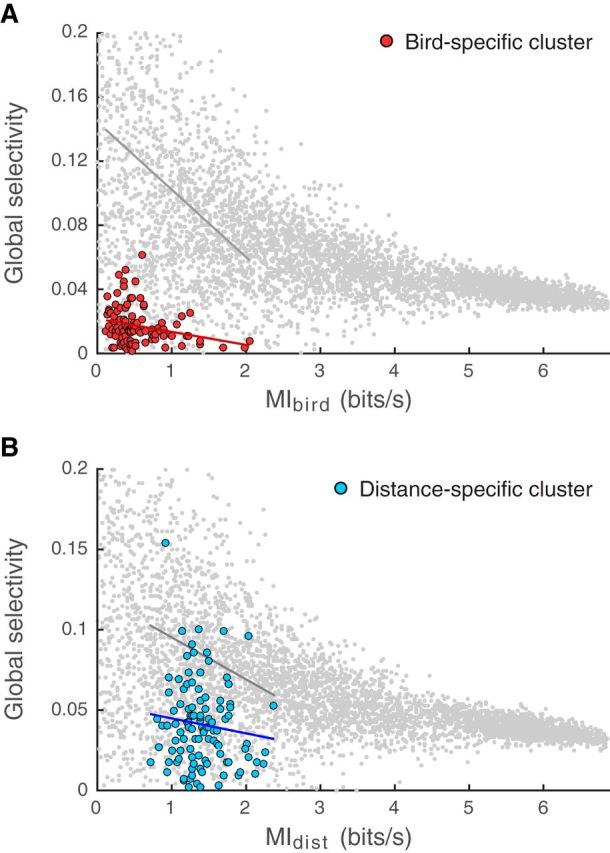
*GS* of bird-specific units (***A***) and distance-specific units (***B***) as a function of the discrimination of individuals (*MI_bird_*) and distances (*MI_dist_*), respectively. Selectivity is quantified using *GS*, where 1 corresponds to maximal selectivity and 0 to no selectivity (see Materials and Methods). Colored points indicate data points. Gray points indicate values obtained from model data (random) with similar information but randomly exploring the range of confusion matrices that could yield this information (see Materials and Methods). Solid lines indicate linear fits to the data. The fit for the model data is restricted to the same range as the fit performed for the actual data. The actual data are restricted to those obtained with *Natural* stimuli only.

We first investigated the units' selectivity: is the high performance of some units due to strong discrimination abilities for certain calls pertaining to a few particular individuals or distances (i.e., a high selectivity), or to a capacity to discriminate between calls pertaining to all individuals or distances (low selectivity)? The *GS* is an entropy-based measure that captures the nonuniformity of the distribution of probabilities of correctly classifying each individual or distance in the confusion matrix (the probabilities sitting in the diagonal of confusion matrices, see Materials and Methods). Figure 11 illustrates the values of *GS* found for both clusters: we show the *GS* calculated from confusion matrices collapsed per bird as a function of the corresponding *MI_bird_* value, for units in the bird-specific cluster (Fig. 11*A*), and the *GS* calculated from confusion matrices collapsed per distance as a function of the corresponding *MI_dist_* value, for units in the distance-specific cluster (Fig. 11*B*). For both clusters, *GS* values are lower than expected by chance for confusion matrices of the same size and of similar values of mutual information (linear models predicting *GS* from the mutual information and the type of confusion matrix: bird-specific cluster *GS* = 0.02 ± 0.0011 vs *random GS* = 0.10 ± 0.0003, *F*_(1)_ = 320, *p* < 10^−6^; Distance-specific cluster *GS* = 0.05 ± 0.0025 vs *random GS* = 0.09 ± 0.0002, *F*_(1)_ = 29.4, *p* < 10^−6^), indicating a low selectivity of the units for particular categories of calls (individuals or distances). Moreover, the effect size was larger for the bird-specific cluster (−2.51) than for the distance-specific cluster (−1.34), further illustrating that the high level of discrimination for the vocalizer identity achieved by the bird-specific cluster units was not the result of high selectivity for particular vocalizer(s).

Thus, whereas previous studies (Elie and Theunissen, 2015) have shown that higher discrimination performance of a particular call type could be associated with a higher selective performance for that specific call type, our results show that high vocalizer discrimination performance is associated with low selectivity for specific individuals or distances. Schneider and Woolley (2013) showed that auditory units could have various levels of selectivity for individuals along the auditory pathway, with some units in NCM having higher values of selectivity compared with units in other auditory regions. However, this analysis was based on songs and not distance calls, and using firing rates and not temporal response patterns. In our data, we observed only small increases in selectivity (but lower information) with rate codes over temporal codes (data not shown).

### Temporal resolution of the neural code

We found that the average spike rate calculated over stimulus presentations for each unit was related to the discrimination performance. Cells showing higher firing rates yielded higher discrimination performances, this being true for *MI_tot_* (Fig. 7) as well as for information for both individuals and distances (*MI_bird_*: *r* = 0.44; for *MI_dist_*: *r* = 0.48, for both: *p* < 10^−6^, *n* = 1322). Next, we refined our investigation of the time code so as to determine whether individual or distance discrimination depended more on the spike pattern or the spike rate.

**Figure 12.**
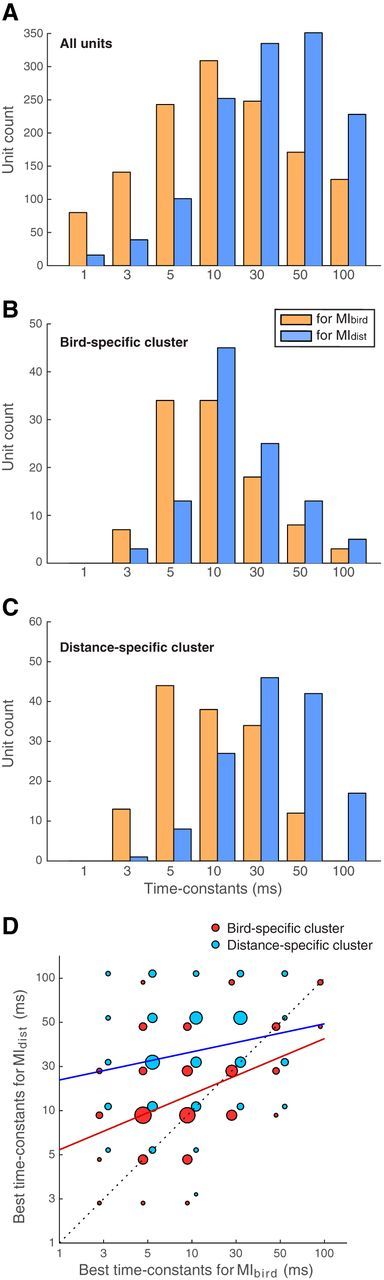
Distribution of the time constants yielding higher discrimination performances. ***A***, For each unit, the time constant (used for the calculation of spike trains' pairwise distances) that yielded the highest individual (*MI_bird_*) or distance (*MI_dist_*) discrimination was selected from a set of 7 values ranging from 1 to 100 ms. ***B***, ***C***, Same representation for the bird-specific cluster and distance-specific cluster, respectively. ***D***, Distribution of the units from both clusters as a function of the time constants yielding the highest *MI_bird_* (*x*-axis) and *MI_dist_* (*y*-axis). The size of each circle represents the number of units corresponding to its coordinates, normalized by the total number of units in each cluster. Jitter along both axes has been added for better visibility. The regression lines calculated for each cluster are represented in red (bird-specific cluster) and blue (distance-specific cluster). Dotted line indicates *y* = *x*. All values are shown using responses to *Natural* stimuli only.

As explained in Materials and Methods, the time constant used to calculate the differences between spike patterns was chosen so as to maximize the discrimination performance as measured by the mutual information of each unit. Seven values were tested as time constants: 1, 3, 5, 10, 30, 50, and 100 ms. Figure 12*A* shows the distribution of the optimal time constant values across all units for the discrimination of vocalizers (*MI_bird_*, in orange) and the discrimination of distances (*MI_dist_*, in blue). As can be seen in the figure, the time constants yielding maximal discrimination performance were shorter for the discrimination of vocalizers (average time constant and standard error of the mean τ = 25.6 ± 0.8 ms) than for the discrimination of distances (40.5 ± 0.9 ms; paired-sample *t* test: *t*_(1321)_ = 13.16, *p* < 10^−6^). We further analyzed the relationship between the time constant values yielding maximal discrimination of individuals and distances for the bird-specific and the distance-specific clusters (Fig. 12*B–D*). To do so, we performed, in addition to *t* tests, a one-way analysis of covariance using a general linear model framework (ANCOVA), with the time constants yielding maximal *MI_dist_* values as the dependent variable, the time constants yielding maximal *MI_bird_* values as a covariate, and the cluster type (bird- or distance-specific) as a factor. Time constant values yielding the highest *MI_bird_* and *MI_dist_* were positively correlated regardless of the cluster (*F*_(1,243)_ = 28.55, *p* < 10^−6^). Figure 12*B*, *C* shows the distribution of the two types of optimal time constant values for the bird-specific and distance-specific clusters, respectively. Both clusters replicated the result found for all the units, the time constants optimal for *MI_dist_* being longer than those optimal for *MI_bird_* (for bird-specific cluster: average time constant and SEM for *MI_bird_* τ = 17.0 ± 1.9 ms and for *MI_dist_* τ = 23.3 ± 2.2 ms; paired-sample *t* test: *t*_(103)_ = 4.25, *p* = 4.64 × 10^−5^; for distance-specific cluster: average time constant and SEM for *MI_bird_* τ = 16.0 ± 1.2 ms and for *MI_dist_* τ = 39.0 ± 2.3 ms, paired-sample *t* test: *t*_(140)_ = 11.02, *p* < 10^−6^). Comparing both clusters, the ANCOVA revealed that the bird-specific cluster is associated with smaller time constants for *MI_dist_* (with a difference between both clusters of 20.7 ms at the intercept; *F*_(1,239)_ = 26.69, *p* < 10^−6^). The interaction between the factor and the covariate was not significant, indicating that there was no discernable difference between the slopes of the two regression lines in Figure 12*D* (*p* = 0.14): time constants yielding maximal *MI_bird_* values were not significantly different between clusters (two-sample *t* test: *t*_(243)_ = 0.25, *p* = 0.80).

Hence, we found a clear distinction in the nature of the neural code for distance versus vocalizer identity: the information about individuals is found in the spike pattern with a temporal resolution of ∼10 ms, whereas the information for distance is in the average firing rate estimated over windows of ∼30 ms. Moreover, although most units carry information about both distance and caller identity, a number of units are also specialized as reflected by the units in the bird-specific and distance-specific clusters. Some of this specialization can be understood in terms of time constants since bird-specific neurons have smaller integration windows for distance coding, these values being closer to the smaller ones used for identity coding. Finally, for all neurons, multiplexing (i.e., coding both identity and distance) can be achieved using a smaller integration window to extract individual information and a longer temporal window to extract distance information. The two temporal windows are not completely independent but are slightly correlated.

### Anatomical properties of the neural units

**Figure 13.**
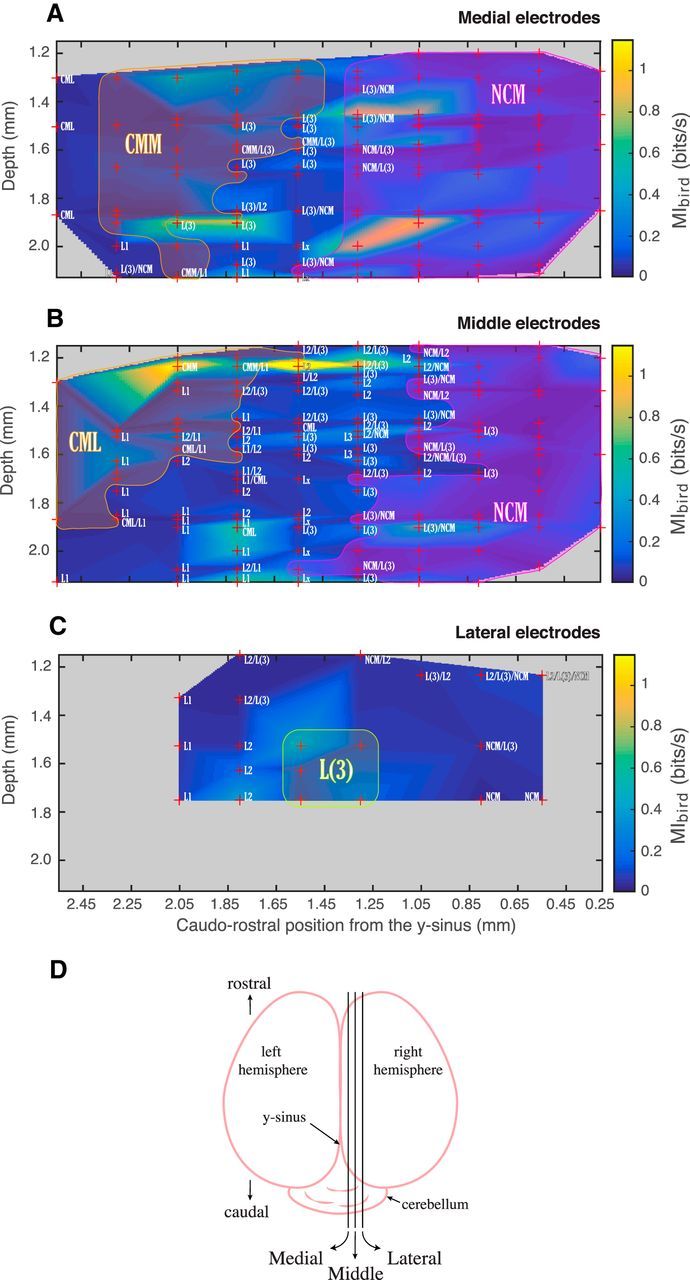
Histological localization of recording sites and individual discrimination for (***A***) medial electrodes (0.3–0.6 mm from midline), (***B***) middle electrodes (0.7–1.1 mm from midline), and (***C***) lateral electrodes (1.2–1.5 mm from midline). Red crosses represent the localization of each recording site for all tested subjects for which histological localization was possible (6 of 8 total), as a function of depth and caudorostral distance from the *y*-sinus. For each recording site, the mean *MI_bird_* value between the corresponding units is calculated, and the graph is constructed by interpolating these values. Color scale represents *MI_bird_*. All values are shown using responses to *Natural* stimuli only. The histological area is given for each recording site localization; when histological results from different birds yielded different area identifications and/or when recording sites were at boundaries, all possible areas are given as a list from more to less probable. Areas where the labels are unique and form contiguous regions are shaded and marked with a single region label (e.g., CMM, CML, or NCM). ***D***, Schematic representation of the localization on the brain (here as an example on the right hemisphere) of the three mediolateral groups of electrodes, determined depending on their distance to the brain's midline (medial, 0.3–0.6 mm; middle, 0.7–1.1 mm; lateral, 1.2–1.5 mm).

**Figure 14.**
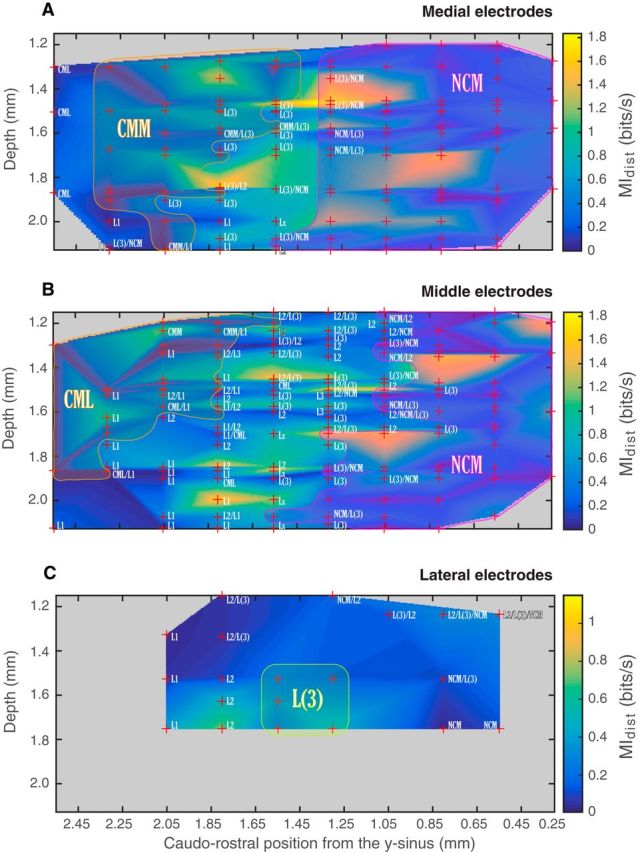
Histological localization of recording sites and discrimination of distance for (***A***) medial electrodes (0.3–0.6 mm from midline), (***B***) middle electrodes (0.7–1.1 mm from midline), and (***C***) lateral electrodes (1.2–1.5 mm from midline). Red crosses represent the localization of each recording site for all tested subjects for which histological localization was possible (6 of 8 total), as a function of depth and caudorostral distance from the *y*-sinus. For each recording site, the mean *MI_dist_* value between the corresponding units is calculated, and the graph is constructed by interpolating these values. Color scale represents *MI_dist_* (all values are shown using responses to *Natural* stimuli only). The histological area is given for each recording site localization; when histological results from different birds yielded different area identifications and/or when recording sites were at boundaries, all possible areas are given as a list from more to less probable. Areas where the labels are unique and form contiguous regions are shaded and marked with a single region label (e.g., CMM, CML, or NCM).

Next, following histological procedures, we assigned each electrode site to either one anatomical area or, in borderline zones, to a number of areas (2 or 3), from the most to least probable (according to our histological analysis). On the one hand, discrimination performance was not significantly different in the different overall coarse anatomical areas of the avian auditory system: CM, NCM, and Field L. We also did not find differences within CM (CLM vs CMM) or within Field L. On the other hand, we did observe reproducible patterns of variability for vocalizer discrimination and for distance discrimination along the dorsoventral axis. To visualize and quantify this effect, we represented the mean values of mutual information (*MI_bird_* and *MI_dist_*) for each recording site across subjects as a function of depth and caudorostral position, for each of the three mediolateral groups of electrodes previously defined depending of the electrodes' distance to the midline (medial, middle, and lateral; see Materials and Methods; Fig. 13*D*). Figure 13 illustrates the localization of recording sites and the average values of their units' vocalizer discrimination performances (*MI_bird_*), whereas Figure 14 illustrates the localization of recording sites and the average values of their units' distance discrimination performances (*MI_dist_*). When comparing visually these two figures, one can observe that, whereas units yielding higher values of *MI_dist_* are more diffuse in their localization, units yielding higher *MI_bird_* values seem to be distributed more focally, either in more superficial or deeper regions of the auditory areas. To determine whether the difference between these two maps of discrimination was statistically significant, we performed various randomizations. We first examined whether the maps of discrimination for distance and for vocalizers were different or correlated. For this purpose, we calculated for each mediolateral group the correlation between the pixel matrices obtained using *MI_bird_* and *MI_dist_* (e.g., for Fig. 13*A* vs 14*A*) and used a permutation analysis (see Materials and Methods) to assess whether it was different from what would be expected by chance. We found a significant positive correlation between the values of mutual information for individuals and distances (medial: *r* = 0.51, *p* < 10^−3^; middle: *r* = 0.21, *p* = 0.045; lateral: *r* = 0.58, *p* = 0.028). Thus, we could not find any major segregation in the localization of individual-oriented versus distance-oriented discriminating units using a 2D representation. Then, to investigate this matter further and to bring out any differences in the units' distribution that could pertain to either the caudorostral or the dorsoventral axes (see Materials and Methods), we projected the MI values on either of these axes and recalculated correlations and significance values. We found that the correlation values calculated from the projections on the caudorostral axis were positive and significant, indicating that there was no fundamental difference in the caudorostral position of units showing high discrimination abilities for individuals or distances (medial: *r* = 0.82, *p* = 0.003; middle: *r* = 0.81, *p* < 10^−3^; lateral: *r* = 0.76, *p* = 0.056). Conversely, we found very low and nonsignificant correlations using the projections on the depth axis for the medial and middle groups, supporting our observation that units showing high individual discrimination abilities are distributed in different areas than the units showing high distance discrimination abilities, the former being mostly distributed in superficial as well as deeper regions of the auditory areas (medial: *r* = 0.08, *p* = 0.74; middle: *r* = 0.04, *p* = 0.87; lateral: *r* = 0.72, *p* = 0.13).

**Figure 15.**
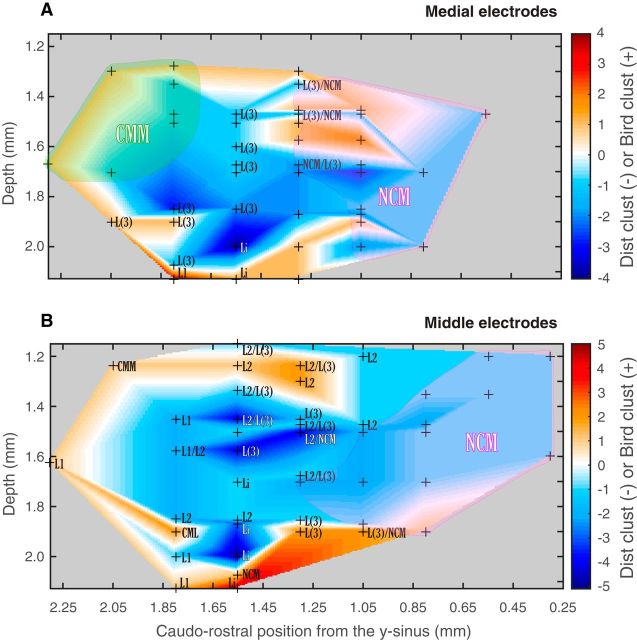
Histological localization of the bird-specific and distance-specific clusters for (***A***) medial electrodes (0.3–0.6 mm from midline) and (***B***) middle electrodes (0.7–1.1 mm from midline). There is no graph for the lateral electrodes, as only two data points were available. Black crosses represent the localization of each recording site for all tested subjects for which histological localization was possible (6 of 8 total), as a function of depth and caudorostral distance from the *y*-sinus. For each recording site, we calculated the number of units corresponding to both clusters, assigning a value of 1 for each unit in the bird-specific cluster and a value of −1 for each unit in the distance-specific cluster. Color scale represents the resulting value for each recording site, and the graph is constructed by interpolating these values. The histological area is given for each electrode localization; when histological results from different birds yielded different area identifications and/or when recording sites were at boundaries, all possible areas are given as a list from more to less probable. Areas where the labels are unique and form contiguous regions are shaded and marked with a single region label (CMM and NCM).

As a last step, and to illustrate closely the differences in the localization of the bird-specific and distance-specific cluster units, we plotted in the same space the distribution of the units from both clusters, assigning for each electrode site a value of 1 for each unit from the bird-specific cluster and a value of −1 for each unit from the distance-specific cluster. Figure 15*A* shows the distribution of both clusters in the medial group, and Figure 15*B* shows their distribution in the middle group. As only two units from the clusters were found in the lateral group, no figure is available for this group. Figure 15 confirms that bird-specific units are mostly found in the medial superficial auditory regions (medial group: CMM, superficial NCM and L/L3, at a depth of ∼1.3–1.6 mm), the more lateral superficial auditory regions (middle group: CMM, L2, at a depth of ∼1.2–1.4 mm), as well as the deep auditory regions (both groups: L1, L/L3, NCM, at a depth of ∼1.9–2.24 mm). Conversely, distance-specific units are mostly found at intermediate depths (from ∼1.5 to 2 mm).

Thus, although we cannot link specific traditional auditory areas to the discrimination of individuals or distances in propagated calls, bird-specific units are mostly found in either superficial or deep regions of the auditory areas and distance-specific units are mostly found at intermediate depths.

## Discussion

Although birds (Klump, 1996; Mathevon et al., 2008; Mouterde et al., 2014a) as well as many other animals (e.g., Mercado and Frazer, 1999) excel at recognizing the information embedded in communication signals degraded by propagation, the neural mechanisms mediating this task remained unexplored. We found neurons in the avian auditory forebrain displaying high discrimination for the identity of unfamiliar vocalizing birds, with remarkable invariance to the sound degradation induced by long-range propagation through the natural environment. This robust neural discrimination may constitute the neural underpinnings of the vocal recognition of social partners in difficult natural settings, a biological process of critical importance for the species survival (Zann, 1984, 1996). One of the main goals of neuro-ethological research is to link neural processes with corresponding behavioral manifestations. Wang et al. (2007) have shown that behavioral performance for song discrimination is correlated with the performance of the most discriminating higher-level single neurons, which activity might potentially be reflecting the computations performed by the entire network. Using behavioral tests, we had previously found that female zebra finches were able to discriminate between the degraded calls of male zebra finches at up to 128 m without training and up to 256 m with training (Mouterde et al., 2014a). Here we found that single neurons could discriminate among unfamiliar individuals with calls that were degraded up to 256 m. Thus, there is an approximate match between single neuron performance and behavioral performance, with single neurons even outperforming behavioral tests in the sense that recognition at the longest propagation distance in behavioral testing was only achieved after repeated training. The neural discrimination of propagated familiar or learned calls might be even superior to the one reported here (Jeanne et al., 2011). Moreover, our neural data were obtained in urethane-anesthetized animals. Although some studies have shown limited effects of urethane on spike rates and discrimination for vocalizations in the primary auditory forebrain of zebra finches (Narayan et al., 2006), other studies in starlings have shown a decrease of selectivity for species-specific sounds (Karino et al., 2016). Thus, neural discrimination values obtained in anesthetized birds might be lower than those obtained in awake behaving animals.

Although it is known that animals (Naguib and Wiley, 2001) and humans (Zahorik, 2002) can assess the distance of sound sources, previous research on the neural substrate of spatial localization has mainly focused on the perception of azimuthal acoustic space (Salminen et al., 2009; e.g., Miller and Recanzone, 2009). Studies about the neural encoding of distance per se are scarce and focused on short-range propagation (Graziano et al., 1999), or target distance in echolocating bats (O'Neill and Suga, 1982). Here, we found that some avian auditory forebrain neurons encoding caller identity can also code the distance separating the receiver and the emitter birds. The aptitude of birds to range conspecifics (Mathevon et al., 2008) may be explained by this neuronal code for distance.

To discriminate the vocal signatures of the propagated calls, neurons had to be invariant to sounds altered in multiple ways. First, the intensity of the signal and the *SNR* decreased with increasing distances, with the *SNR* becoming negative at 128 m. Second, the spectrotemporal structure of the signal was modified: the frequency band with an *SNR* >0 dB was progressively reduced with distance to a narrow band at ∼3.5 kHz; the spectral modulations, such as those observed in harmonic stacks, lost their sharpness; and, similarly, the temporal envelope of the sound was smeared in time (Mouterde et al., 2014b). Thus, to maintain neural discrimination for vocalizers, auditory neurons must be invariant not only to intensity changes but also to background noise regardless of the signal strength and to changes in temporal and spectral sharpness. Previous studies, also using zebra finches, have described invariance properties of song-responsive neurons, either to intensity (Billimoria et al., 2008) or to background noise (Moore et al., 2013; Schneider and Woolley, 2013). Here, neurons showing high vocalizer discrimination achieve high discrimination performance at low intensities in noise, yielding robust responses for a large range of *SNR* values. We predict that the most distance invariant neurons would have nonlinear spectrotemporal receptive fields that are more sensitive to spectral structure ∼3.5 kHz and exhibit a lack of sensitivity to rapid temporal modulations in the envelope and to overall intensity (see also Moore et al., 2013). Testing encoding models that combine nonlinear intensity-responses curves or gain adaptation (Rabinowitz et al., 2012) with linear spectrotemporal receptive fields could be performed in future work to validate this prediction.

We further investigated the highly discriminating neurons in terms of their coding properties, their putative cell type, and their anatomical localization. In terms of coding timescale, the information about individuals is found in spike patterns on a shorter integration window (∼10 ms), whereas the information about distance is in the firing rate estimated over longer windows (∼30 ms). The fact that the best discrimination for identity is found with short time windows corroborates numerous studies showing that neurons use a spike timing strategy to encode vocalizations (Wang et al., 2007; Huetz et al., 2011; Gaucher et al., 2013; Elie and Theunissen, 2015) and is consistent with what was found previously for neural discrimination of conspecific song in the Field L of zebra finches (Narayan et al., 2006). These spike patterns might originate from processing a particular sequence of acoustic features, but additional neuronal mechanisms must be in place to preserve this information despite the natural variations in these acoustic features.

In contrast to the optimal timescale for individual discrimination, the optimal timescale for distance discrimination is noticeably higher. This difference in coding timescales allows most neurons to carry information about distance and caller identity, each temporal window being used to extract the corresponding category of information, in what is also called temporal multiplexing. Temporal multiplexing has been shown in a number of studies using mammalian models (e.g., for carrying complementary information in visual or somatosensory perception; Panzeri et al., 2010) as well as in auditory perception (Kayser et al., 2009; Walker et al., 2011). Multiplexing enhances the coding capacity of the system, enabling disambiguation of stimuli that cannot be discriminated at a single response timescale, and makes sensory representation stable in regard to variability (Kayser et al., 2009).

Our results also support the idea that the neural processes underlying perceived distance do not depend solely on simple features but might involve higher-level cortical processing to integrate various aspects of propagation-induced degradation (Wightman and Kistler, 1993; Zahorik et al., 2005).

Beyond studying the nature of the neural code, it is also important to begin to describe neural circuits that generate this code (Kayser et al., 2009; Panzeri et al., 2010). Here we examined the anatomical location and putative cell types of both bird- and distance-specific neurons. A critical substrate for the analysis of auditory scenes has been found in Field L in the form of intensity invariant neurons (Billimoria et al., 2008). Additionally, noise invariant neurons were found in the NCM (Moore et al., 2013; Schneider and Woolley, 2013), and previous studies have highlighted the importance of secondary auditory areas (NCM and CM) in processing higher-order features, such as behavioral significance, and their role in learned auditory discrimination, such as individual recognition (Chew et al., 1996; Gentner, 2004; Menardy et al., 2012). In this study, we did not find that the neural substrate for the discrimination of individuals or distances was linked to specific traditional auditory areas, but we did find a distinctive spatial distribution of these neurons, the units most discriminative of individual identities being mostly found in superficial and deep regions of the auditory areas, whereas the units most discriminative of distance are more likely to be found at intermediate depths. Such dorsoventral functional organization has also been described previously in NCM, both for coding complex sound features (Ribeiro et al., 1998) as well as for noise invariant tuning (Moore et al., 2013). Concerning the neuron type, we found that units most discriminative of individual identity or distance consisted of a majority of narrow-spiking neurons. This might appear to be in contrast with previous reports where higher selectivity based on spike rates has been associated with broad-spike types (Nagel and Doupe, 2008; Meliza and Margoliash, 2012; Schneider and Woolley, 2013; Yanagihara and Yazaki-Sugiyama, 2016). We found the opposite result by analyzing the neural code based on spike patterns and quantifying selectivity using information measures (or decoding performance): highly discriminating neurons are principally composed of narrow-spike types, which have higher firing rates and higher information rates. Thus, there seems to be a compromise between high selectivity based on spike rate differences and found in broad-spike units, and high discrimination based on temporal codes found in narrow-spike units.

In conclusion, we have provided evidence that single neurons in the avian forebrain can perform the computations needed to both identify and range a behaviorally relevant auditory source. It remains to be determined how further gains in neural discrimination of both identity and range could be achieved by examining the ensemble code. Summing responses across all neurons (also called “population response”) could provide a robust neural signal for call intensity and range regardless of the caller identity. Additional ensemble codes, such as temporal coherence, could further help create stable auditory objects for spectrally and temporally complex and varying natural sounds (Shamma et al., 2011). The exploration of neural assemblies, rather than single neurons (Kayser et al., 2009; Quian Quiroga and Panzeri, 2009), of attentional mechanisms in awake behaving birds and of the plasticity of perception occurring with experience or learning (Dahmen and King, 2007) will further help us understand how the complex task of scene analysis is performed by the brain.
